# How recreational marathon runners hit the wall: A large-scale data analysis of late-race pacing collapse in the marathon

**DOI:** 10.1371/journal.pone.0251513

**Published:** 2021-05-19

**Authors:** Barry Smyth

**Affiliations:** Insight SFI Research Centre for Data Analytics, School of Computer Science, University College Dublin, Dublin, Ireland; University of Rome, ITALY

## Abstract

**Introduction:**

In the marathon, how runners pace and fuel their race can have a major impact on race outcome. The phenomenon known as *hitting the wall (HTW)* refers to the iconic hazard of the marathon distance, in which runners experience a significant slowing of pace late in the race, typically after the 20-mile mark, and usually because of a depletion of the body’s energy stores.

**Aim:**

This work investigates the occurrence of significant late-race slowing among recreational marathoners, as a *proxy* for runners hitting the wall, to better understand the likelihood and nature of such slowdowns, and their effect on race performance.

**Methods:**

Using pacing data from more than 4 million race records, we develop a pacing-based definition of hitting the wall, by identifying runners who experience a sustained period of slowing during the latter stages of the marathon. We calculate the *cost* of these slowdowns relative to estimates of the recent personal-best times of runners and compare slowdowns according to runner sex, age, and ability.

**Results:**

We find male runners more likely to slow significantly (hit the wall) than female runners; 28% of male runners hit the wall compared with 17% of female runners, *χ*^2^(1, *N* = 1, 928, 813) = 27, 693.35, *p* < 0.01, *OR* = 1.43. Such slowdowns are more frequent in the 3 years immediately before and after a recent personal-best (PB) time; for example, 36% of all runners hit the wall in the 3 years before a recent PB compared with just 23% in earlier years, *χ*^2^(1, *N* = 509, 444) = 8, 120.74, *p* < 0.01, *OR* = 1.31. When runners hit the wall, males slow more than females: a relative slowdown of 0.40 vs. 0.37 is noted, for male and female runners, when comparing their pace when they hit the wall to their earlier race (5km-20km) pace, with *t*(475, 199) = 60.19, *p* < 0.01, *d* = 0.15. And male runners slow over longer distances than female runners: 10.7km vs. 9.6km, respectively, *t*(475, 199) = 68.44, *p* < 0.01, *d* = 0.17. Although, notably the effect size of these differences is small. We also find the finish-time costs of hitting the wall (lost minutes) to increase with ability; *r*^2^(7) = 0.91, *p* < 0.01 *r*^2^(7) = 0.81, *p* < 0.01 for male and female runners, respectively.

**Conclusions:**

While the findings from this study are consistent with qualitative results from earlier single-race or smaller-scale studies, the new insights into the risk and nature of slowdowns, based on the runner sex, age, and ability, have the potential to help runners and coaches to better understand and calibrate the risk/reward trade-offs that exist as they plan for future races.

## Introduction

In the marathon, terms such as “hitting the wall” (HTW), “bonking”, or “blowing up” refer to the sudden onset of debilitating fatigue that can occur late in the race. At best, this can temporarily slow even the most accomplished and experienced runners, but it can also render a runner unable to muster much more than a walking pace for the remainder of the race and may prevent some from finishing. While most marathon runners are familiar with the notion of hitting the wall—many even claim to have experienced it in person [1, 2]—it should be recognised that truly hitting the wall is not the same as the feeling of generalized fatigue and discomfort that is part and parcel of running the marathon distance [3–5]. The conventional wisdom is that runners hit the wall when their glycogen stores become depleted, usually as a result of poor race nutrition [6–9], which can be exacerbated by aggressive pacing [7, 10, 11], and there is thought to be an important cognitive component too [12, 13]. While experienced marathoners understand how to avoid hitting the wall, it remains a significant risk among recreational marathoners, especially novices and first-timers.

The central objective of this work is to explore the nature of these slowdowns by analysing more that 4 million race-day records; the scale of this study distinguishes it from much of the work on hitting the wall that has come before [1, 2, 11, 14, 15]. We identify runners who suffer significant and sustained slowing during the latter stages of the marathon, and examine the characteristics of these slowdowns (frequency, start, duration, degree, finish-time cost) in relation to sex, age, and ability.

We find male runners to be much more likely than female runners to hit the wall [11, 14], regardless of age or ability, and we find that slowdowns occur more frequently in the years immediately before and after a recent personal best. Moreover, when males hit the wall, they slow more than female runners, and over longer distances. Although the costs of these slowdowns (lost minutes) are broadly similar between males and females, they tend to increase with ability, with faster runners experiencing a greater finish-time cost than slower runners.

## Related work

The phenomenon of hitting the wall is perhaps the most iconic hazard of the marathon distance, but a similar effect can be found in other endurance events too, including ultra-marathons, adventure races, cycling and the triathlon. Fortunately, the most catastrophic examples of hitting the wall remain relatively rare, but the phenomenon continues to impede many marathoners, especially less experienced recreational runners. And despite the significance of the phenomenon, consensus has yet to be reached on a precise conceptual or operational definition; see [15, 16]. It is usually framed as a *fatigue* and *fueling* problem [7, 17, 18]: simply put, if an athlete runs out of the energy they need to fuel their remaining race, then they will have to slow or even stop. However, the relationship between fatigue and performance is not a straightforward one, and the topic continues to be a source of debate in the literature. In what follows, we review related work on fatigue, pacing and performance, as it relates to the phenomenon of hitting the wall in the marathon, in order to frame the work presented in this study.

### Fatigue & fueling

Historically, fatigue can mean different things to different disciplines [17, 18]: a physiologist might view fatigue as the failure of a specific physiological system [19]; biomechanists may view it in terms of a decrease in the force output of muscles [20, 21]; while a sports psychologist will typically view fatigue as the ‘feeling’ of tiredness [22, 23]. It is not surprising, therefore, that research into fatigue-induced changes in exercise performance involves several different disciplines and perspectives, and has led to the development of several different models to explain the fatigue response that arises from prolonged exercise.

For example, Noakes [17] and Green [19] discuss how the *cardiovascular/anaerobic* model assumes that fatigue occurs when the cardiovascular system is no longer able to supply the necessary oxygen to, or remove waste products from, the working muscles; see also [24]. A related model is the *energy supply/energy depletion* model [17, 19, 25], which proposes that fatigue is the result of two mechanisms: (1) a failure to provide sufficient ATP to the working muscles, via the various metabolic pathways; and (2) a fueling problem, due to the depletion of fuel substrates, namely muscle and liver glycogen, blood glucose and phosphocreatine [8, 9].

Alternatively, the *neuromuscular fatigue* model links fatigue with a diminished muscular response to electrical stimulus as a result of prolonged exercise [17, 20, 25–27], while the *muscle trauma* model proposes that fatigue is a consequence of the type of muscle damage [28, 29] that commonly occurs during prolonged exercise (muscle swelling and stiffness, or the tearing of muscle fibres etc.). The *motivational* model of fatigue is based on a lack of interest in exercise performance, akin to losing the will to perform [22, 30, 31]. While it is often incorporated into the neromuscular model of fatigue and the *central governor* model (see below), the motivational model uniquely holds that neuromuscular function is *intentionally* down-regulated, rather than subconsciously altered.

In the *central governor* model Noakes [32], Noakes et al. [33], and Ulmer [34] argue that exercise performance is controlled by a governor located in the central nervous system, which uses signals and feedback from muscles and other organs to regulate exercise performance, in order to protect vital organs from injury or damage. More recently, Lambert et al. [13] and Gibson & Noakes [35] have extended the central governor model by proposing the *complex systems* model of fatigue. This model integrates a variety of peripheral signals and sources of feedback, in a non-linear manner, in order to regulate activity to allow for the completion of a given bout of exercise. Accordingly, fatigue is a subconscious sensation that reflects the underlying state of this integrative process.

In marathon running, the phenomenon of hitting the wall is associated with the rapid onset of debilitating fatigue and, as the above viewpoints suggest, it may arise from a combination of factors including inadequate fueling, a lack of training, or a diminished intentional state. Recently Rapoport’s energy model [7] has been developed with marathon running in mind, and it offers an opportunity to predict when a runner will become fatigued based on their energy stores and pace. The model is based on the premise that it takes approximately 1 calorie to move a runner per kilo of body mass and per kilometer of running, regardless of pace [36, 37]. Rapoport’s model extends this by considering: (a) the source of energy—fat vs. carbohydrates—with per-km energy expenditure varying, not by pace, but by the source of the energy; and (b) the amount of carbohydrates available. Romijn et al. [38] discuss how faster runs are fueled by a greater proportion of carbohydrates than fat. Whether a runner will hit the wall depends on how quickly their glycogen stores deplete, which Rapaport found depends on a combination of a runner’s aerobic capacity (or *VO*_2_ *max*), the density of muscle glycogen, and the relative mass of their leg musculature. Hagen et al. [39] report that a higher aerobic capacity leads to a faster marathon, provided there are adequate glycogen stores, while Fairchild et al. [40] note that larger leg muscles, relative to body mass, are associated with a higher percentage of *VO*_2_ *max* that can be sustained, because a lower body mass means a lower running energy cost, and larger leg muscles mean more room to store glycogen. The utility of this model is that it can be used to estimate the distance at which runners will exhaust their glycogen stores as a function of pace, thereby providing a basis for optimising the performance of endurance runners and predicting mid-race fueling needs.

In conclusion, fatigue is an inevitable consequence of the marathon distance, and the need for in-race fueling is a necessary response to the natural limits of the human body’s energy stores. Together, fatigue and depleting energy reserves can conspire to dramatically slow even the swiftest runner, when they hit the wall, and, in what follows, we will consider the further implications of this for pacing and performance.

### Pacing and performance

Pacing in endurance events is an important research topic, particularly when it comes to understanding the optimal pacing strategy for a given event type. For example, Tucker et al. [41] examined the pacing strategies of male runners in world-record performances to show how pacing strategies varied with distance. Shorter events were characterised by fast starts, followed by progressive slowing, while 5,000m and 10,000m events were associated with fast starts and fast finishes, with a period of slower running during the middle of the race. March et al. [42] conclude that more even pacing tends to be associated with faster finish-times in the marathon, with females associated with more consistent pacing than males, even when the effects of ability and age were controlled for [43–46]. Tucker & Noakes [47] emphasise how pacing can be impacted by many different factors. For instance, the work of Trubee [48] found that pacing difference between the sexes increased with temperature; see also the work of Cuk et al. [49].

Smyth [10] examined more than one million marathon race records, of mostly recreational runners, to explore the relationship between starting and finishing paces, and overall race performance, in the marathon. The conventional wisdom is that starting too fast can create pacing problems later in the race—including hitting the wall—but, equally, finishing too fast may signal that a runner has paced too conservatively. Starting or finishing too fast was found to be associated with slower overall finish-times, as partly predicted by Denison [50]. Indeed, fast starts were found to be especially injurious to performance, in part because they increased the likelihood that a runner would go on to hit the wall later in the race.

More recently, the work of Oficial-Casado et al. [51] considered differences in pacing profiles in four big-city marathons (Valencia, Chicago, London, and Tokyo) to find that differences between corresponding sections of these races tended to increase with finish-time increases. In particular, the pacing of the first 5km of the races analysed differed significantly, with London having the fastest first 5km and also the greatest difference in relative speed between the first and second half of the race. These results, underscore pacing differences that can exist between races and highlight the importance of accounting for race pacing characteristics when selecting a marathon and a suitable pacing strategy.

### On the psychology & phenomenology of hitting the wall

Despite what is known about how runners pace their races, the related phenomenon of hitting the wall appears to be less well understood. One reason for this might be because the phenomenon remains relatively rare among elite runners—the usual targets of performance studies—even though many recreational marathoners do confront it at some stage in their marathon history [1, 2, 12].

Some of the literature that does exist focuses on the perceptions, expectations, and cognitive orientations of runners who hit the wall. For example, one early study by Summers et al. [2] surveyed 363 middle-aged, recreational, first-time marathoners to evaluate their reasons for attempting the marathon, their perceived outcomes from the event, and their experiences during the race. Overall, 56% of respondents reported hitting the wall, with just over 73% of them experiencing it after the 19 mile (30km) mark. In related work by Stevinson & Biddle [1], the focus was on the relationship between a recreational runner’s cognitive orientation and hitting the wall. The 66 participants (56 males and 10 females) in this study were all entrants into the 1996 London marathon, and the sample included 35 marathon first-timers. Of the 53% who reported hitting the wall—more males than females—they were much more likely to adopt a cognitive orientation of ‘inward distraction’ and a sense of internal disassociation as they attempted to distance themselves from the task at hand.

Buman et al. [11] produced a more in-depth study of the phenomenologcial characteristics of hitting the wall, based on a survey of 315 runners, to assess whether they felt they had hit the wall and, if so, their perceptions of 24 different characteristics linked to the experience. Once again, a high proportion (43%) of respondents reported hitting the wall and the study concluded that four characteristics—generalised fatigue, unintentional slowing, a desire to walk, and a shifting focus on survival—were especially salient. However, surprisingly, only 70% of those who reported hitting the wall also reported a concomitant slowdown. In related work, Buman et al. [14] looked at the relationship between the risk profile of runners and when they are likely to hit the wall, in order to describe the overall functional form of risk over the course of a marathon. The sex of a runner, their training volume, and their race expectations were found to play important roles in predicting whether someone would be likely to hit the wall, with the risk peaking at mile 21 followed by a steep subsequent decline; see also [1, 12].

These studies provide useful reference rates for hitting the wall among recreational runners, although it seems unwise to conclude that more than 40–50% of all recreational runners will actually hit the wall in a given race, in practice. It is more likely that the methodology used by these studies might elicit an over-reporting of the phenomenon, especially if many less experienced runners conflated the usual late-race feelings of fatigue, and a natural slowdown, with the idea of hitting the wall. If there was no material deterioration in pace for up to 30% of those who claimed to have hit the wall as per Buman et al. [11], then it seems doubtful that they actually did experience the phenomenon. Indeed, if hitting the wall is seen as a *rite of passage* for marathoners, then using the phenomenon to justify a disappointing performance may prove to be all too tempting and common. An alternative explanation for the lack of a reported slowdown could be that some respondents simply did not report the unintentional slowing of pace as a major factor, even though it did occur. Either way, the potential objectivity shortcomings of these self-reporting studies speak to the additional value that may be provided by a more evidence-based pacing study, such as the one presented here.

## Data & methodology

This study is based on an original dataset of marathon race records. All of the data is publicly available from the corresponding marathon websites and a complete list of URLs of these web-sites is provided in S1 Table in the supporting information to this article. The research was approved as being exempt from a full ethical review by the Human Research Ethics Committee (Sciences) at University College Dublin on the grounds that it involves the anonymous analysis of public data. This section describes this dataset in detail, explains the approach used to determine when a runner hits the wall, and discusses how this can be used to compare runners who hit the wall based on their sex, age, and ability.

### Datasets

The data for this study was incrementally collected between 2015 and 2019. The resulting dataset includes 4,183,362 race records for an estimated 2,743,322 unique runners, from 270 races that took place in 38 cities during the period from 2005 to 2019. Each race record is associated with a runner name, age information, and an indication of whether a runner was male or female. We refer to this as the *original* dataset. For reasons discussed below, the main analysis in this study is conducted on a subset of this original dataset, by focusing on runners who are associated with multiple race records. We refer to these as *repeat runners* and to their data subset as the *repeaters* dataset. This subset contains 2,179,221 race records (approximately 52% of the race records from the original dataset) for 717,940 unique runners (approximately 26% of the original dataset’s unique runners).

#### The original dataset

The original dataset includes marathons that provide timing data for 5km race segments (0–5km, 5–10km, …, 35–40km, plus the final 40–42.195km segment); the requirement for 5km segments is based on the need to track changes in pacing during different stages of the marathon. Note that we refer to each 5km segment by its end-point, thus the 10–15km segment is the *15km* segment; the exception is the shorter 40–42.195km segment, which is called the *final* segment. This means that each complete race record includes 9 separate segment times.

The type of age information provided varies from marathon to marathon. Sometimes precise age (or year of birth) information is included, but often it is limited to age ranges or categories. To maximise the availability of age information across the entire dataset, in this study we rely on the following age ranges, 20–39, 40–44, 45–49, 50–54, 55–59, 60+, which are either directly available from, or can be derived for, all of the race records in the original dataset.

Summary details of this original dataset are presented in Table 1 for each marathon, showing the number of participants, the percentage of female participation, the mean and standard deviation of finish-times (mins), and the percentage of participants who are deemed to have hit the wall, based on the definition developed below. In addition, a further summary table is provided by Table 2 showing similar data based on age group.

**Table 1:** A summary of the original dataset by city/race showing the city name and years for which there are data, the number of runners (#*R*), the percentage of female runners (%*F*), the average finish-time (*FT*) and the percentage of runners hitting the wall (%*HTW*) based on the operational defintion adopted here.

City (years)	#R	%F	FT (mins)	%HTW
Amsterdam (2012–2019)	90,644	23.78	250.41 ± 38.48	28.25
Athens (2013–2019)	88,366	19.41	282.44 ± 52.68	29.78
Aukland (2017–2018)	3,037	27.96	268.09 ± 51.15	41.72
Austin (2017–2019)	7,537	79.83	283.90 ± 53.67	38.11
Barcelona (2011–2019)	114,612	16.22	240.26 ± 35.19	29.65
Berlin (2010–2019)	346,047	26.60	253.73 ± 42.52	22.25
Boston (2010–2019)	230,414	46.84	239.61 ± 41.69	29.81
Chicago (2005–2019)	537,496	45.67	277.80 ± 52.48	39.05
Cologne (2016–2019)	18,206	23.15	250.58 ± 36.73	31.47
Copenhagen (2011–2019)	74,035	24.87	248.79 ± 35.24	36.50
Dubai (2016–2019)	7,401	22.70	274.75 ± 49.51	54.63
Eindhoven (2008–2019)	17,488	17.48	232.30 ± 29.46	29.47
Frankfurt (2005–2019)	139,772	20.69	242.75 ± 36.08	26.31
Gold_Coast (2008–2018)	49,733	35.03	261.44 ± 47.00	51.54
Hamburg (2013–2019)	79,241	23.27	246.17 ± 36.03	17.90
Helsinki (2015–2017)	7,806	28.20	263.42 ± 39.68	29.93
Honolulu (2016–2018)	27,898	43.59	340.64 ± 62.33	58.84
Houston (2011–2018)	54,595	38.59	271.21 ± 43.79	34.55
Los Angeles (2015–2019)	77,578	41.58	311.26 ± 62.49	47.04
London (2011–2019)	322,337	39.64	276.49 ± 54.86	37.72
Madrid (2013–2019)	71,242	10.79	249.62 ± 36.49	42.99
Melbourne (2015–2018)	21,523	46.94	252.97 ± 38.60	53.02
Mexico (2013–2017)	82,534	26.38	308.39 ± 53.18	67.65
Moscow (2017–2019)	24,353	17.11	251.15 ± 39.70	25.51
New York (2006–2019)	564,537	39.08	272.58 ± 50.13	34.62
Oslo (2016–2018)	6,425	24.76	243.01 ± 35.07	31.97
Paris (2014–2019)	240,330	24.25	266.33 ± 49.94	39.12
Prague (2016–2018)	18,545	22.77	258.97 ± 44.30	43.15
Rome (2018–2019)	19,293	50.57	264.03 ± 47.05	39.52
Rotterdam (2012–2019)	88,214	21.94	249.52 ± 35.29	36.80
Singapore (2016–2018)	20,789	17.07	348.94 ± 49.64	78.36
Stockholm (2005–2019)	200,366	24.92	256.81 ± 39.15	24.41
Sydney (2013–2018)	18,678	29.03	253.61 ± 39.07	40.46
Tokyo (2015–2018)	119,436	22.00	293.16 ± 59.71	46.95
Valencia (2014–2018)	63,936	15.27	236.97 ± 32.81	25.97
Vienna (2005–2019)	82,840	18.31	243.32 ± 34.82	21.01
Warsaw (2015–2018)	24,629	17.51	249.95 ± 37.14	32.95
Washington (2008–2018)	221,449	43.13	290.38 ± 51.28	47.81

**Table 2:** An overall summary of the original dataset by age group, showing the number of male (*M*) and female (*F*) runners in each age group, with their average finish-times, and the percentage of male and female runners deemed to have the wall, based on the definition adopted in this work.

	Number of Runners		Finish-Time (mins)		%HTW	
Sex	F	M	F	M	F	M
Age Group						
20–39	748,336	1,229,479	281.08 ± 51.53	256.57 ± 48.83	27%	43%
40–44	224,067	487,120	280.48 ± 50.79	254.26 ± 47.76	23%	37%
45–49	183,468	449,870	282.41 ± 49.71	253.97 ± 46.34	23%	35%
50–54	117,967	320,580	289.8 ± 50.14	260.76 ± 47.96	24%	36%
55–59	56,634	185,202	297.79 ± 50.91	268.62 ± 48.51	25%	37%
60+	34,253	146,386	314.82 ± 52.67	288.76 ± 52.68	26%	39%

#### The repeaters dataset

The repeaters dataset is summarised in a similar manner in Table 3. It includes runners with more than one race record in the original dataset. The reason for this is that our analysis of how runners hit the wall relies on an estimate of their ability and we use an estimate of their recent personal-best time for this. As above, Table 4 shows these statistics based on age group.

**Table 3:** A summary of the repeaters dataset by city/race showing the city name and years for which there are data, the number of runners (#*R*), the percentage of female runners (%*F*), the average finish-time (*FT*) and the percentage of runners hitting the wall (%*HTW*) based on the operational defintion adopted here.

City (years)	#R	%Rpt	%F	FT (mins)	%HTW
Amsterdam (2012–2019)	40,219	44.37	21.21	244.67 ± 37.45	20.05
Athens (2013–2019)	35,008	39.62	12.81	276.00 ± 51.27	22.63
Aukland (2017–2018)	999	32.89	26.03	258.15 ± 49.24	25.32
Austin (2017–2019)	2,575	34.16	76.61	277.78 ± 52.44	22.08
Barcelona (2011–2019)	40,095	34.98	13.81	233.49 ± 33.49	19.02
Berlin (2010–2019)	192,450	55.61	24.70	249.15 ± 41.70	15.77
Boston (2010–2019)	139,871	60.70	46.39	233.79 ± 38.52	22.23
Chicago (2005–2019)	338,197	62.92	44.36	269.84 ± 51.73	29.09
Cologne (2016–2019)	10,252	56.31	22.04	245.78 ± 36.42	22.84
Copenhagen (2011–2019)	38,405	51.87	22.93	242.78 ± 33.69	25.98
Dubai (2016–2019)	3,019	40.79	22.11	260.64 ± 46.59	37.70
Eindhoven (2008–2019)	8,268	47.28	16.35	227.43 ± 28.62	19.03
Frankfurt (2005–2019)	97,851	70.01	20.18	239.30 ± 35.23	18.41
Gold_Coast (2008–2018)	23,268	46.79	32.00	247.87 ± 42.95	33.44
Hamburg (2013–2019)	49,388	62.33	21.93	242.41 ± 35.47	13.08
Helsinki (2015–2017)	3,737	47.87	25.83	257.90 ± 39.43	22.04
Honolulu (2016–2018)	5,406	19.38	39.51	315.10 ± 63.63	40.99
Houston (2011–2018)	39,430	72.22	38.09	265.72 ± 43.59	26.21
Los Angeles (2015–2019)	46,992	60.57	41.47	303.90 ± 62.68	35.24
London (2011–2019)	95,383	29.59	35.70	257.97 ± 51.81	23.61
Madrid (2013–2019)	50,892	71.44	10.97	244.95 ± 35.30	27.51
Melbourne (2015–2018)	7,329	34.05	37.83	248.19 ± 37.11	40.33
Mexico (2013–2017)	29,513	35.76	23.43	298.18 ± 53.27	51.71
Moscow (2017–2019)	6,281	25.79	12.93	241.52 ± 36.72	14.75
New York (2006–2019)	326,560	57.85	38.26	264.69 ± 48.77	24.49
Oslo (2016–2018)	3,160	49.18	27.37	236.93 ± 33.02	14.92
Paris (2014–2019)	117,079	48.72	22.01	259.04 ± 48.24	29.19
Prague (2016–2018)	6,841	36.89	22.58	252.87 ± 42.56	30.82
Rome (2018–2019)	5,353	27.75	36.29	261.77 ± 46.12	32.15
Rotterdam (2012–2019)	45,328	51.38	20.35	243.34 ± 34.41	27.31
Singapore (2016–2018)	7,016	33.75	15.32	343.91 ± 50.30	63.58
Stockholm (2005–2019)	142,598	71.17	23.93	252.67 ± 38.92	18.16
Sydney (2013–2018)	8,544	45.74	27.51	245.58 ± 37.45	26.10
Tokyo (2015–2018)	23,013	19.27	22.43	278.82 ± 59.06	32.28
Valencia (2014–2018)	25,012	39.12	14.03	232.84 ± 32.21	17.28
Vienna (2005–2019)	25,863	31.22	14.43	237.58 ± 33.64	13.93
Warsaw (2015–2018)	8,137	33.04	14.87	244.82 ± 37.38	24.68
Washington (2008–2018)	129,889	58.65	43.18	283.94 ± 51.23	35.16

**Table 4:** An overall summary of the repeaters dataset by age group showing the number of male (*M*) and (*F*) runners in each age group along with their average finish-times and the percentage of male and female runners hitting the wall, based on the defintion adopted here.

	Number of Runners		Finish-Time (mins)		% HTW	
Sex	F	M	F	M	F	M
Age Group						
20–39	296,717	469,414	269.09 ± 48.69	246.37 ± 45.2	17%	30%
40–44	109,930	244,567	270.88 ± 48.3	245.84 ± 44.38	15%	27%
45–49	94,540	239,527	273.97 ± 47.33	246.91 ± 43.82	15%	26%
50–54	61,781	176,515	281.12 ± 47.67	253.4 ± 45.44	16%	26%
55–59	30,617	104,001	289.37 ± 48.38	262.02 ± 46.43	16%	27%
60+	18,922	82,282	306.93 ± 50.3	282.59 ± 51.45	17%	29%

We identify repeaters by matching race records based on a combination of a runner’s name identifier, sex, and age. Precise age information (or year of birth) is used when available, otherwise age ranges are used. Infrequently, this approach incorrectly matches runners with the same name, age, and sex, who are competing in a single race and such ambiguous matches are excluded. This approach is estimated to be sufficient to identify a large fraction of legitimate repeaters from the original dataset.

### An operational definition of hitting the wall

For the purpose of this study, we determine a runner to have hit the wall if they experience significant slowing for an extended period during the second half of the race; this is similar to the pacing-based definition of hitting the wall developed by Berndsen et al. [15]. Obviously, this is an imperfect measure of whether a runner truly hits the wall. It will both overestimate and underestimate the true number who hit the wall; for example, some runners will slow due to injury or lack of training/fitness, rather than because they genuinely hit the wall, while others may hit the wall too late in the race to be identified. Nevertheless, this approach should be sufficient to provide an estimate that is good enough to use at scale in this analysis.

More formally, we determine a runner *r* to have hit the wall (Eq 1) when they slow by at least a factor of *s* –– their *degree of slowdown* or *DoS* –– for at least *d* kms –– their *length of slowdown* or *LoS* –– after the 20km mark; that is, for segments from the 25km segment (20–25km) to the final segment. To calculate a runner’s degree of slowdown for a given race segment we use their *base-pace* (*BP*) as a reference pace. Their base-pace is their average pace during the 5km–20km portion (that is, the 3 × 5*km* segments, 5–10km, 10–15km, and 15–20km) of the race, as shown in Eq 2; we exclude the initial 5km segment because pacing during the very early stages of the marathon tends to be more erratic [10] as it can take time for runners to *locate* their desired pace after a congested start. Then, the degree of slowdown is based on the ratio of their second-half segment paces to their base-pace, as shown in Eq 3. For example, if *r* has a base-pace of 5 mins/km and they slow to 6 mins/km during the 30–35km segment, then their degree of slowdown for this segment will be 0.2. Finally, we can calculate the length of a slowdown (*LoS*) as the sum of the distances for any second-half segments in which the runner slows by at least *s*; see Eq 4.

$$\begin{array}{c} HTW(r,s,d)\Leftrightarrow LoS(r,s)\;\geq \;d \end{array}$$ (1)

$$\begin{array}{c} BP\left(r\right)=\sum_{10km\;\leq \;seg\;\leq \;20km}pace(r,seg)/15 \end{array}$$ (2)

$$\begin{array}{c} DoS\left(r,seg\right)=\frac{1-pace(r,seg)}{BP(seg)} \end{array}$$ (3)

$$\begin{array}{c} LoS\left(r,s\right)=\sum_{\forall seg\;\geq \;25km\;:\;DoS(r,seg)\;\geq \;s}distance(seg) \end{array}$$ (4)

To better understand the relationship between the fraction of runners hitting the wall, according to this definition, and the *DoS* and *LoS* thresholds, we conduct a *sensitivity analysis* to evaluate different ranges for these parameters. We use the full original dataset for this particular analysis, since it does not rely on repeat runners, and the results inform the selection of suitable *DoS* and *LoS* values to use in the remainder of our analysis.

### Runner ability & the cost of hitting the wall

For runner ability we use an estimate of a runner’s recent personal-best time (PB). More formally, if *races*(*r*, *y*) is the set of race records for runner *r* that are within 3 years of year *y*, then we estimate *r*’s *ability* (in year *y*) as their fastest race-time in these recent races; see Eq 5.

$$\begin{array}{c} PB\;Time\left(r,y\right)=\min_{m\;\in \;races(r,y)}Finish\;Time\left(m\right) \end{array}$$ (5)

$$\begin{array}{c} HTW\;Cost\left(r,m_{h}\right)=HTW\;Time\left(m_{h}\right)-PB\;Time\left(r,year\left(m_{h}\right)\right) \end{array}$$ (6)

$$\begin{array}{c} Relative\;HTW\;Cost\left(r,m_{h}\right)=\frac{HTW\;Time\left(m_{h}\right)-PB\;Time\left(r,year\left(m_{h}\right)\right)}{PB\;Time(r,year\left(m_{h}\right)} \end{array}$$ (7)

It is important to note that this estimate of a runner’s recent PB time may not be their true recent PB time, if their PB race is missing from our dataset; we discuss this further when we consider the limitations of this study. These recent PB times are also used to estimate the cost of hitting the wall (Eq 6), by calculating the difference between a runner’s finish-time, when they hit the wall (*HTW Time*), and their recent *PB Time*; see Eq 5. For example, if a runner achieves a finish-time of 275 minutes when they hit the wall, and if their recent PB is 235 minutes, then we estimate the cost of hitting the wall to be 40 minutes, or a *relative cost* of 0.17 indicating a 17% finish-time loss; see Eq 7.

### Research questions

Using the repeaters dataset, we compare runners based on their sex, age range, and ability level (estimated PB time in 30-minute intervals), to answer the following research questions, using the metrics defined above:

1. What proportion of runners hit the wall (*HTW Proportion*) in a given race? We do this by calculating the proportion of male and female runners who hit the wall (based on Eq 1) for each age range and ability level.
2. How does the proportion of runners hitting the wall vary in the years before and after a runner achieves a PB? We answer this by calculating the proportion of male and female runners hitting the wall based on the number of years before and after achieving their *overall* fastest finish-time (estimated PB).
3. If a runner hits the wall, then when does their slowdown begin (*HTW Start*), how long is it sustained for (*HTW Distance*), and by how much do they slow (*HTW Slowdown*)? We answer this by calculating the average *HTW Start*, *HTW Distance*, and *HTW Slowdown* metrics for male and female runners who hit the wall for each age group and ability level.
4. What is the finish-time cost (*HTW Cost*) when a runner hits the wall, relative to their recent PB time? We evaluate this by calculating the average *HTW Cost* and *Relative HTW Cost* for each age group and ability level.

### Statistical analysis

We use a combination of unequal variance *t* tests and *χ*^2^ tests of proportions to evaluate the significance of the differences observed between male and female runners (within a given age group or ability level) and to evaluate the significance of the differences observed for male and female runners for successive age groups and ability levels. In each case a significance threshold of *p* < 0.01 is used to determine significance with Cohen’s *d* used to measure effect size for *t* tests and the *odds ratio* (*OR*) for *χ*^2^ tests. Where relevant, we will also use a Wald test with t-distribution as the test statistic, to evaluate if the slope of a linear regression line is different from 0—to evaluate a trend—using a significance threshold of *p* < 0.01 with *r*^2^ as the corresponding effect size. In Figs 2–6 the statistical significance of the results is encoded in the following ways:

- In each graph we show the mean values for male and female runners as horizontal lines. If the difference between these overall means is statistically significant, then these lines are displayed as solid lines, otherwise they are displayed as dashed lines.
- Significant differences between corresponding results for male versus female runners are indicated by filled markers in each result graph. For example, in Fig 2, all of the differences between males and females are judged to be significant (based on a *χ*^2^ test of proportions) for *p* < 0.01, regardless of age or ability; all of the individual markers are filled. In contrast, there is no significant difference between the average *HTW Start* experienced by males and females who are 60 years or older, as indicated by the corresponding unfilled markers in Fig 5(a).
- A solid line connecting two markers on a graph indicates that the (within-sex) difference is statistically significant. For example, in Fig 2(b), the *HTW Proportions* between the 330 and 360-minute ability groups are not statistically significant, for females, as indicated by the dotted line connecting these markers.

The raw data for each result graph and the corresponding statistical analysis results are available as S1 Datasets.

## Results

### Sensitivity analysis

**Fig 1:**
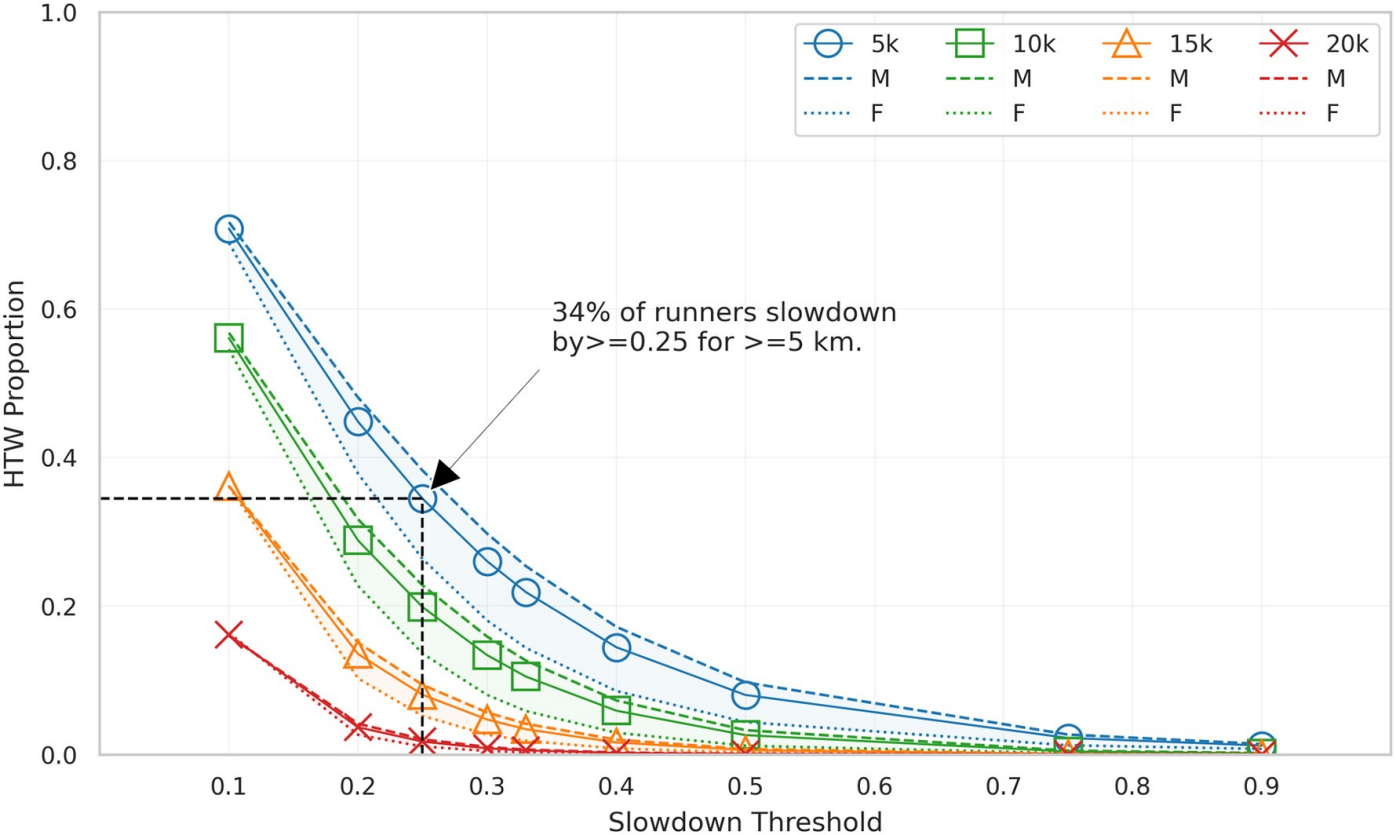
The proportion of runners in the original dataset hitting the wall by slowdown (DoS) and minimum length (LoS) thresholds.

The sensitivity analysis results in Fig 1 show how the proportion of runners hitting the wall changes in a predictable manner for different *DoS* and *LoS* thresholds. As expected, larger slowdowns over longer distances correspond to smaller proportions of runners hitting the wall. For the purpose of this study we define hitting the wall using a slowdown (*DoS*) threshold of 0.25 and a minimum distance (*LoS*) threshold of 5km—that is, runners must slow by at least 25% for at least 5km—which corresponds to 34% of runners in the original dataset hitting the wall, as indicated in Fig 1. These thresholds are comparable with similar thresholds reported by Berndsen et al. [15] where slowdowns of approximately 17% over more than 5km were proposed to identify runners hitting the wall.

This proportion of runners hitting the wall also conforms with reasonable expectations about how many marathoners hit the wall in practice. Although this is lower than the proportions (40–50%) reported by [1, 2, 11] using self-reported, post-hoc surveys of runners, as we shall see in the following section, the proportion of runners hitting the wall depends on ability and more than 40% of male runners with slower PBs do hit the wall based on the definition used here.

Finally, it is worth noting that minor changes in these thresholds do not substantially change the nature of the results. Later, in a discussion of the limitations of this analysis, we will discuss this aspect in more detail and supporting evidence is available in S1–S14 Figs.

### The proportion of runners hitting the wall

**Fig 2:**
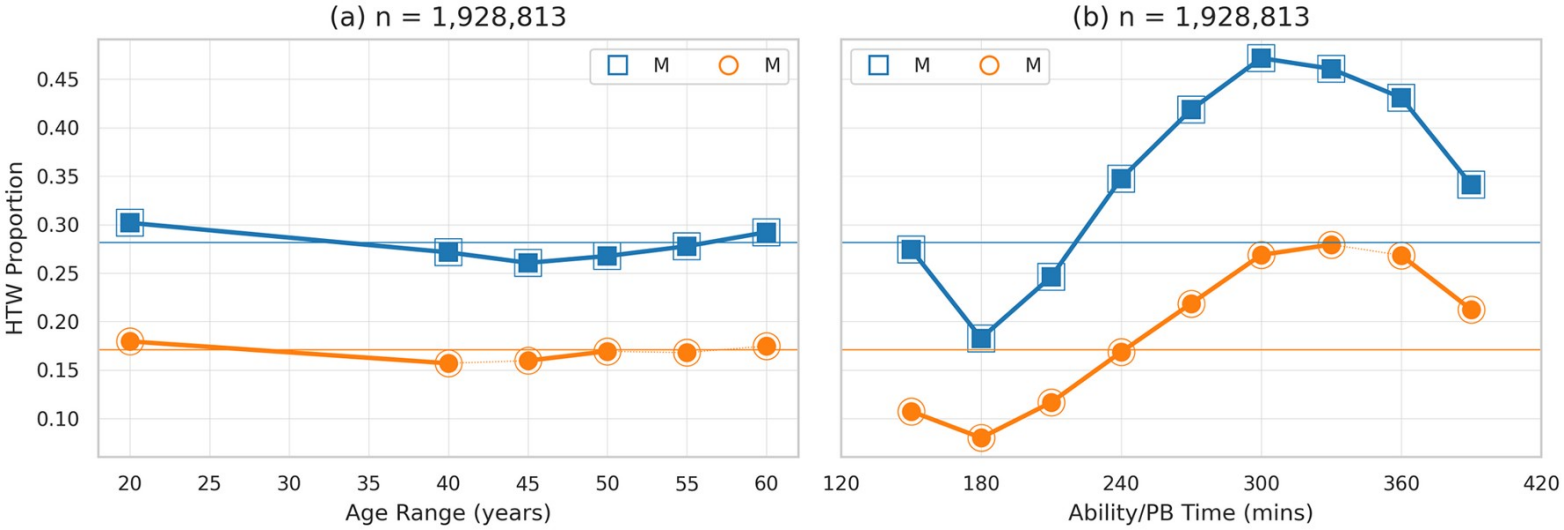
The proportion of male and female runners hitting the wall by (a) age range and (b) ability level.

Fig 2 shows the proportion of runners hitting the wall based on sex, age group, and ability level. Overall 28% of male runners hit the wall compared with only 17% of female runners, *χ*^2^(1, *N* = 1, 928, 813) = 27, 693.34, *p* < 0.01, *OR* = 1.43, and while ability level clearly influences the proportion hitting the wall, age plays a more modest role.

In Fig 2(a) there is evidence that younger runners are more likely to hit the wall, with *HTW Proportions* reaching a low-point for the 45–49 age group. The effect size associated with the differences between males and females remain high for each age group, 1.79 ≤ *OR* ≤ 2.0, while the effect size between successive age groups for males and females is more modest, 0.93 ≤ *OR* ≤ 1.17.

Fig 2(b) shows how the proportion of runners hitting the wall increases steadily with recent PB times between 3 and 5–5.5 hours. All of the differences between males and females, for each ability level, are significant with *p* < 0.01 and 1.9 ≤ *OR* ≤ 3.14 and a majority of the differences between successive (within-sex) ability levels are also statistically significant with *p* < 0.01 and 0.61 ≤ *OR* ≤ 1.69 for males and 0.65 ≤ *OR* ≤ 1.38 for females.

### The likelihood of hitting the wall based on PB year

**Fig 3:**
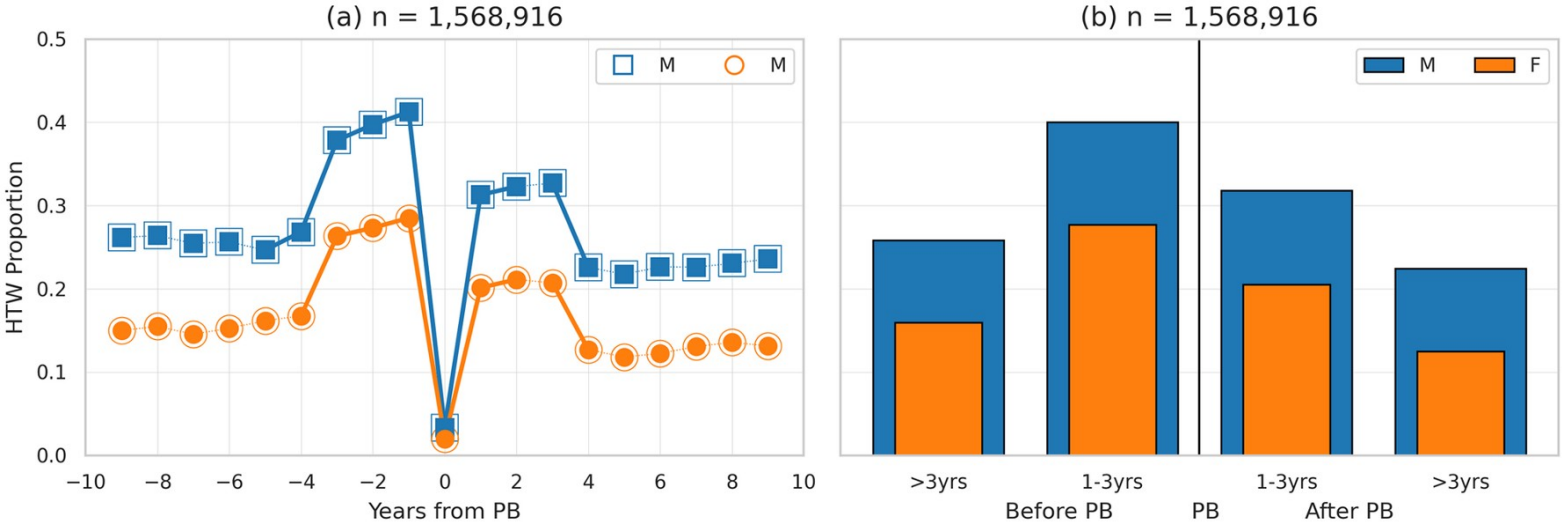
The proportion of male and female runners hitting the wall based on the number of years before (<0) and after (>0) achieving their overall (estimated) PB: (a) shows the relevant proportions of runners hitting the wall for each year before and after a PB; (b) shows the aggregate proportion of runners hitting the wall during four periods, 4–9 years before/after a PB and 1–3 years before/after a PB.

It is also interesting to see how HTW proportions vary in the years before and after a runner achieves their *overall* PB; note, here we are using a runner’s *overall* fastest finish-time in our dataset, rather than the *recent* (3-year) PB, used to determine current ability. In Fig 3(a), races are aligned so that runners achieve their (overall) PB in year 0 and then we calculate the HTW proportions for up to 9 years before and after this PB year; there are of course fewer runners available the farther we move from their PB year, and some runners with more distant races (>9 years from PB) are obviously not included. The results indicate that, in the three years before or after a runner achieves their PB, they are significantly more likely to hit the wall, compared with earlier or later years, respectively.

This is summarised in Fig 3(b), as the aggregate proportion of male and female runners hitting the wall in the 3 years before and after a PB, compared to 4–9 years before and after a PB. For example, 1–3 years before achieving an overall PB, 40% of male runners hit the wall, compared to just under 26% in the 4–9 year period before achieving the PB, *χ*^2^(1, *N* = 338, 057) = 6, 165.03, *p* < 0.01, *OR* = 1.25. Likewise, 28% of female runners hit the wall in the 3 years before a PB compared with 16% in earlier years, *χ*^2^(1, *N* = 171, 387) = 2, 503.39, *p* < 0.01, *OR* = 1.50. A similar result is observed for male and female runners in the years after achieving a PB too.

It is also worth noting that the differences between the proportions of male or female runners who hit the wall in the 1–3 years *before* their PB (40% and 28% for males and females, respectively) is significantly larger that the corresponding proportion of runners hitting the wall in the 1–3 years *after* their PB (32% and 21% for males and females, respectively) with *χ*^2^(1, *N* = 494, 211) = 3, 626.53, *p* < 0.01, *OR* = 1.10 for males and *χ*^2^(1, *N* = 260, 747) = 1, 835.09, *p* < 0.01, *OR* = 1.22 for females.

Thus, proximity to a PB represents a significant risk factor in terms of hitting the wall for male and female runners, and the risk is higher just before achieving a PB than it is just after a PB. This is likely due to more runners adopting more aggressive pacing as they attempt to secure a new PB and we will consider this further in the discussion section of this paper.

**Fig 4:**
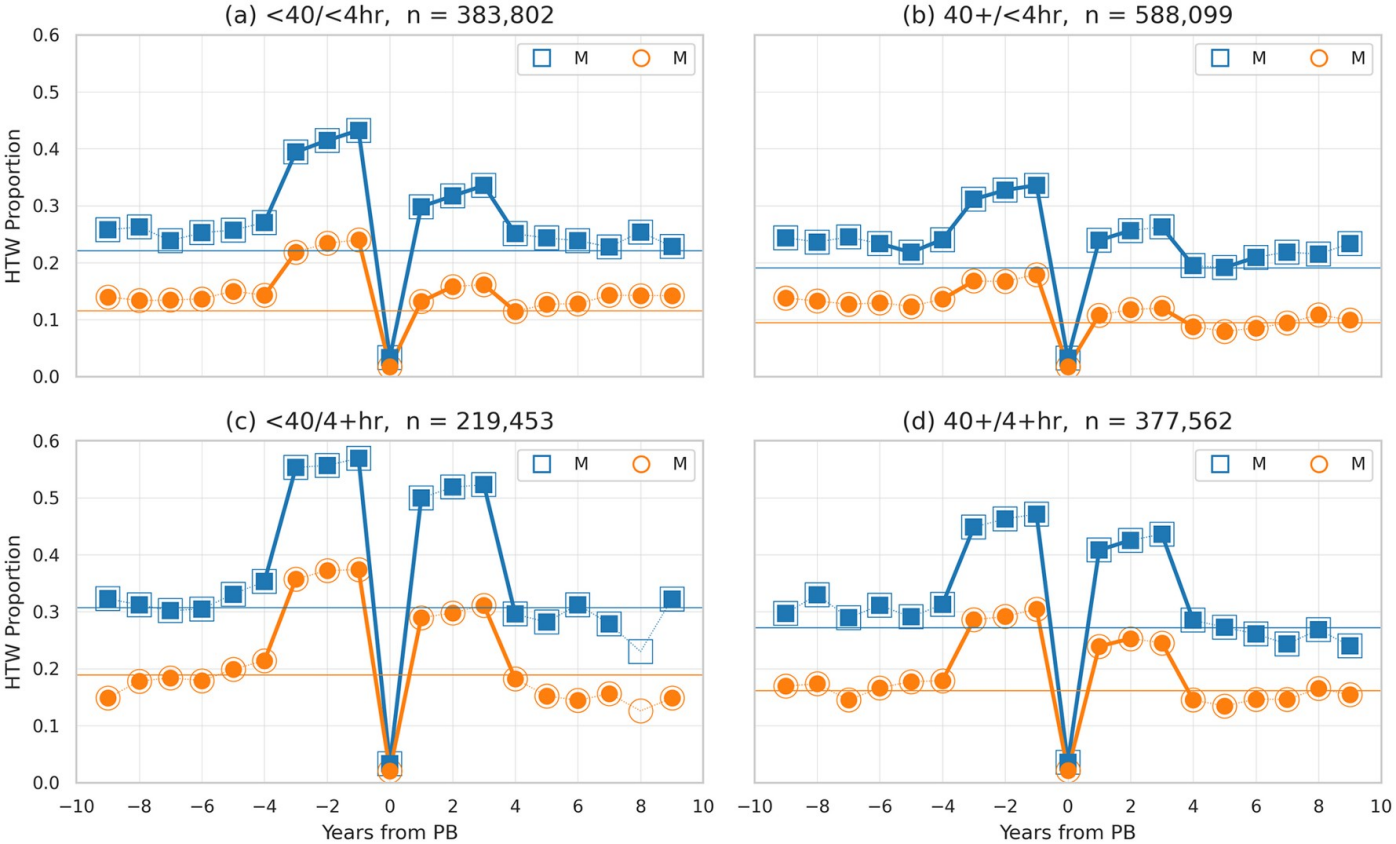
The proportion of male and female runners hitting the wall based on the number of years before (<0) and after (>0) their overall (estimated) PB, and based on age and ability.

For completeness, Fig 4 groups runners based on their age (<40 vs. ≥40) and overall PB times (<4 hours vs. ≥4 hours), to explore whether there is an age or ability effect, when it comes to HTW risk in the years before and after a PB. Similar spikes in *HTW Proportion* are evident in all 4 groupings. Younger (<40 years-old) and slower (≥4 hour finishes) runners are the most at risk in close proximity to a PB; for example, more than 50% of younger and slower male runners hit the wall the year before their PB as per Fig 4(c). On the other hand, older (≥40 years-old) runners with <4 hour finish-times are the least at risk, with the proportion of HTWs peaking at just over 30% for males; see Fig 4(b). Once again we observe a similar pattern of statistically significant differences: (i) a greater proportion of males hit the wall than females in each cohort; (ii) the proportion of runners hitting the wall increases significantly in proximity to a PB; and (iii) the proportion of runners hitting the wall is higher in the 3 years before a PB than in the 3 years after. The full dataset for these results is available in S1 Datasets.

### The dimensions of the wall

**Fig 5:**
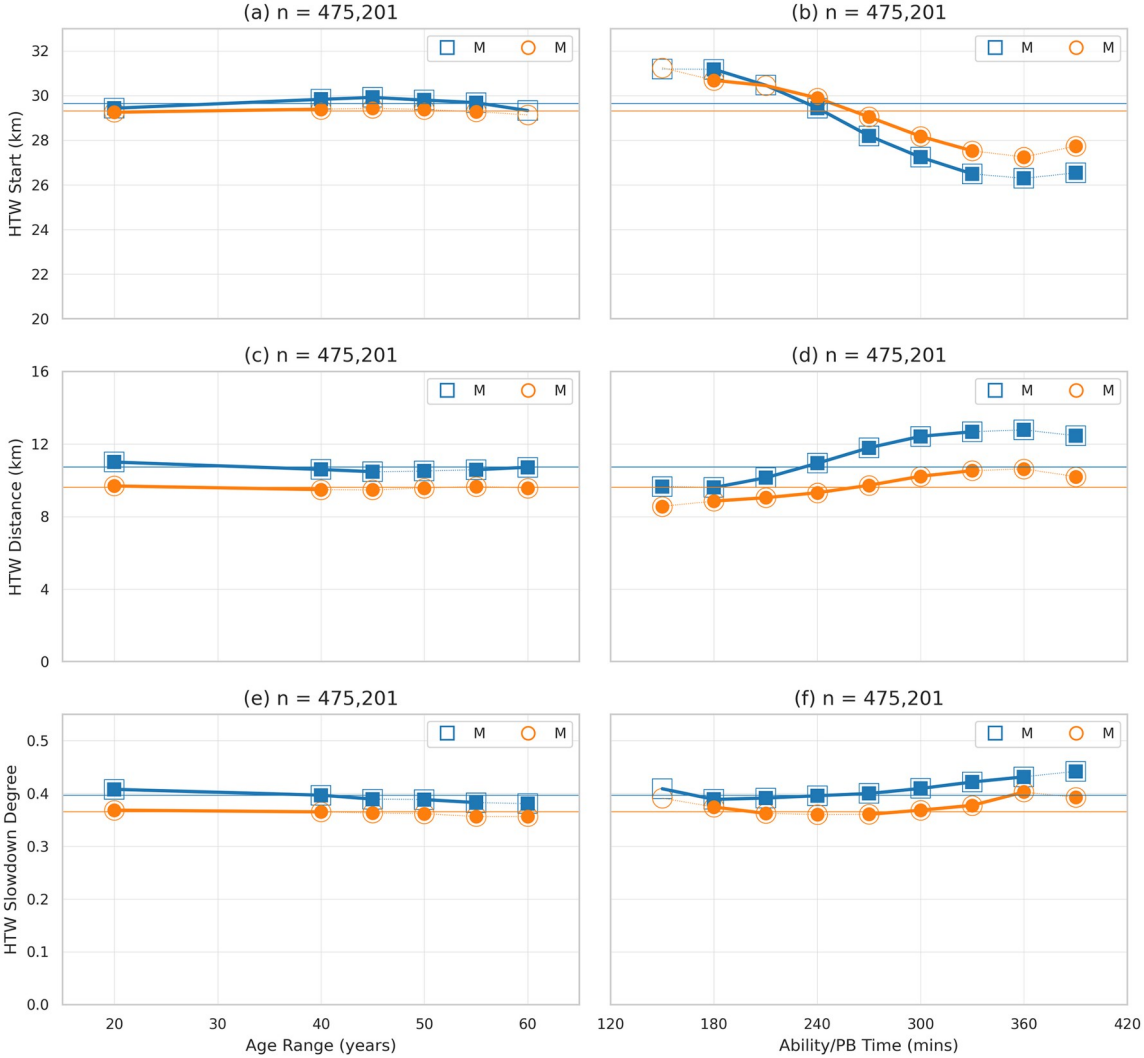
HTW dimensions (start, distance, slowdown) for male and female runners by age range and ability. *HTW Start* refers to the average distance at which runners begin the slowdown that corresponds to their hitting the wall. *HTW Distance* refers to the length of this slowdown and *HTW Slowdown* refers to the degree of this slowdown, relative to their base-pace (that is, their average pace during the 5–20km portion of the marathon).

Fig 5a–5f show the dimensions of the wall in terms of the start of the slowdown (*HTW Start*), the duration or distance (*HTW Distance*) of the slowdown, and degree of the slowdown (*HTW Slowdown*), and how they relate to age and ability for male and female runners. On average male runners begin their slowdown slightly later (29.6km) than female runners (29.3km), *t*(475, 199) = 20.03, *p* <.01, *d* = 0.05. Males sustain their slowdown for longer than females (10.72km vs. 9.61km, respectively), *t*(475, 199) = 68.44, *p* <.01, *d* = 0.17. And, and on average the degree of slowdown for males is 0.40 compared with 0.37 for females, *t*(475, 199) = 60.20, *p* <.01, *d* = 0.15. However, although these are statistically significant differences the effect size is modest (*d* < 0.2).

Fig 5a, 5c and 5e show that age plays a very minor role in terms of the start, distance, and degree of slowdown, but there is a stronger relationship between these metrics and ability. A Wald test confirms a non-zero slope of the regression line between these metrics and estimated PB time, for male and female runners, *r*^2^(7)>0.69, *p* < 0.01, except in the case of the degree of slowdown of female runners (*p* = 0.31). The differences between male and female runners for each ability level are, generally speaking, statistically significant based on Welch’s *t* test (*p* < 0.01) but the mean effect size for *HTW Start* is very small (*d* = 0.10±0.11) compared with *d* = 0.35±0.09 for *HTW Distance* and *d* = 0.20±0.08 for *HTW Slowdown*.

Thus, we can conclude that while a runner’s ability and sex influences how they hit the wall (the start, duration, and degree of slowdown) the differences observed are generally small, with males slowing by a little more, and for slightly longer distances, than females. It is worth noting that this longer distance for males implies that females are more likely to recover from their slowdown before the end of the race, which is consistent with results reported by Smyth [10] showing that females are more likely to finish faster than their mean race-pace than males.

### HTW cost

While it is straightforward to evaluate the finish-time of a runner when they hit the wall, it is less clear what their finish-time would have been had they not. We cannot replay the race without them hitting the wall, for example, but we can at least estimate their lost minutes (*HTW Cost*) by calculating the difference between their finish-times when they do hit the wall (*HTW Time*) and their *recent* estimated PB times, as in Eqs 6 and 7.

**Fig 6:**
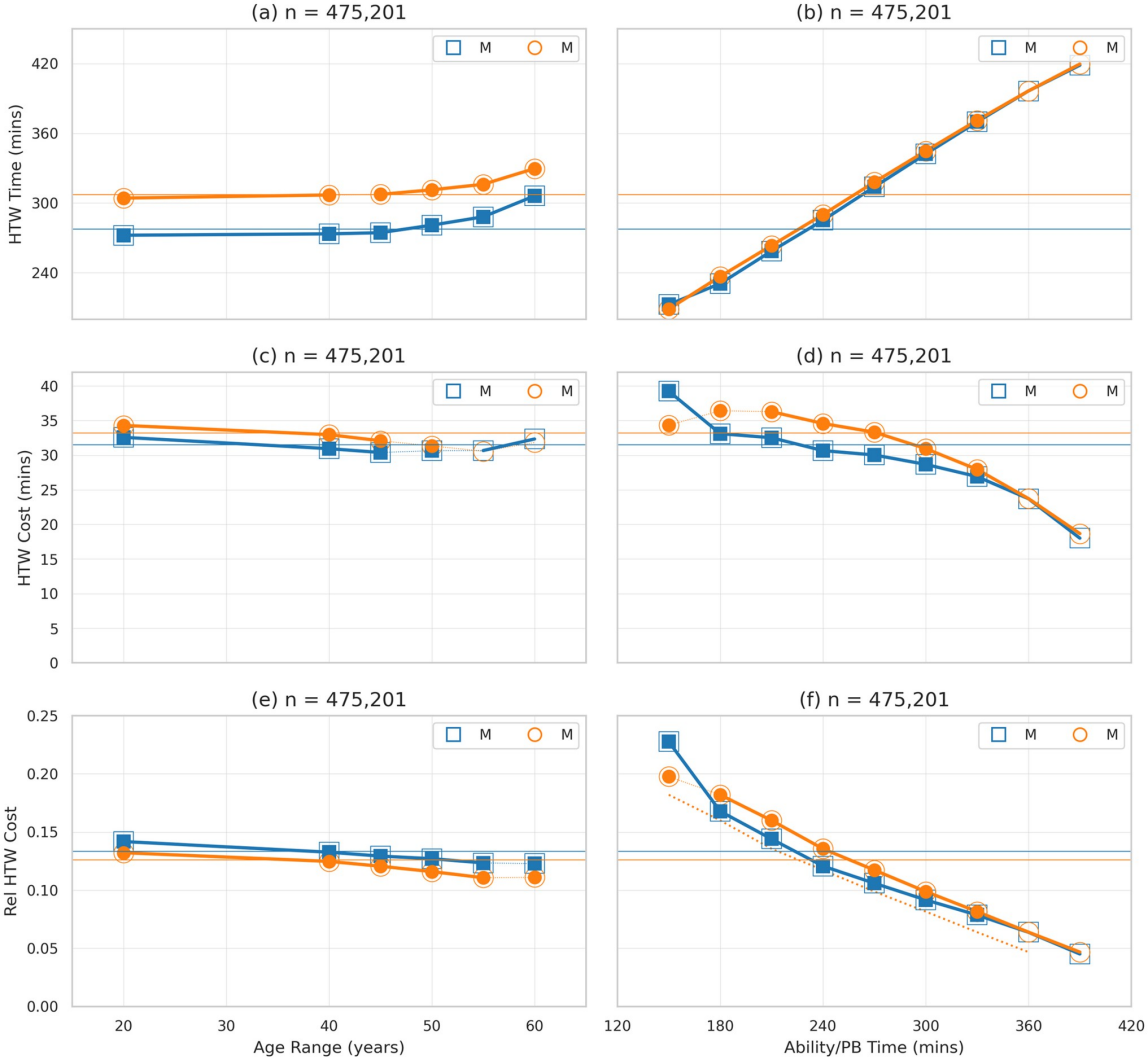
The finish-time and cost associated with hitting the wall for male and female runners by age range and ability. *HTW Time* refers to the finish-time in minutes when a runner hits the wall. *HTW Cost* refers to the difference between a runner’s HTW Time and their estimated PB time. *Rel HTW Cost* refers to a runner’s HTW Cost as a fraction of their PB time.

Not surprisingly, the mean *HTW Time* of males (277.44 minutes) is significantly faster than for females (307.28 minutes), as indicated by the horizontal mean lines in Fig 6a and 6b; *t*(475, 199) = −179.76, *p* < 0.01, *d* = 0.44. In Fig 6(a) we can see that this difference is preserved across all age groups (*d* = 0.65±.08 for these age groups) and how *HTW Time* tends to increase with age, and more noticeably for older runners.

However, these sex differences are less apparent when we group runners by ability (recent PB times) as shown in Fig 6(b); note how the slower mean finish-times of females is accounted for by an increasing number of runners in the slower PB ranges. As expected, HTW times increase monotonically with recent PB times and runners of a given ability tend to experience a similar HTW time when they hit the wall; there continues to be a modest but statistically significant difference between males and females, for each ability level, but the effect size is trivial, *d* = 0.09±0.11.

The cost implications of hitting the wall are shown in Fig 6c–6f. Overall, males suffer from a smaller average finish-time cost than females, 31.50 minutes vs 33.20 minutes, respectively—*t*(475, 199) = −19.78, *p* < 0.01, *d* = 0.05 —but the effect size is clearly very small. However, there is a strong linear relationship between *HTW Cost* and ability; see Fig 6(d). Using a Wald test to confirm a non-zero slope for the linear regression lines we find *r*^2^(7) = 0.91, *p* < 0.01 for males and *r*^2^(7) = 0.81, *p* < 0.01 for females. The relationship is even stronger when we account for the cost of hitting the wall as a fraction of PB time in Fig 6(f), *r*^2^(7) = 0.93, *p* < 0.01 for males and *r*^2^(7) = 0.99, *p* < 0.01 for females.

Thus, faster runners tend to experience a greater finish-time cost than slower runners. However, it must be recognised that this does not mean that faster runners slow by more or for longer than slower runners when they hit the wall. We know from the previous section that slower runners tend to begin slowing earlier and for longer than faster runners, and they slow down by a greater degree too. Thus, the greater finish-time cost experienced by faster runners is due to their proportionally faster PB races, compared with the PBs of slower runners.

It is also worth remarking on the fact that male runners experience a greater *relative* cost than female runners, for a given age group—Fig 6(e)—yet this is not the case when we compare them based on ability, as in Fig 6(f). This is likely due to physiological differences between male and female runners, which are responsible for faster finish-times for the former. It means, for example, that a female runner with a 3-hour PB time is not equivalent to a male runner with a 3-hour PB time; all other things being equal the female runner will be achieving a higher level of relative performance than the male runner. In the past, some researchers have compensated for this by reducing female finish-times [46]. When we apply a 30-minute adjustment—that is, by reducing female times by 30 minutes—then the relative HTW costs for females drop below those of males, as indicated by the dashed line in Fig 6(f); the differences between males and these adjusted female values remain statistically significant. Thus, while there is some evidence to suggest that females experience a greater finish-time cost than males, when they hit the wall, the effect size is very small and complicated by confounding physiological differences between male and female runners.

## Discussion

The results presented here show that male runners are significantly more at risk of hitting the wall than females. This is consistent with the existing literature on pacing differences between male and female runners [43, 45, 52] and on the literature about hitting the wall itself [1, 14]. It can be explained, in part at least, by the tendency of males to take more pacing risks; see for example recent work by Hubble et al. [53], in which male runners were found to consistently overestimate their marathon abilities, leading to more aggressive and risky pacing strategies.

The finding that runners are much more likely to hit the wall in the years directly before a PB appears to be a novel one, and may also be explained by risk-taking behaviour and sub-optimal pacing decisions when runners are *chasing a PB*. This is also consistent with the similar spike in the proportion of runners hitting the wall in the 3 years directly *after* achieving a PB, as some runners continue to try to improve their PB time, perhaps encouraged by their recent PB success. However, the fact that the post-PB spike is significantly less than the pre-PB spike suggests that at least some runners are satisfied to return to safer pacing patterns having achieved a new PB. This highlights the delicate balance that exists between racing hard (to secure a PB) and avoiding pacing problems later in a race, and is consistent with other work on the risks associated with starting a marathon too fast, as reported by Smyth [10], and recent work by Deaner et al. [54] showing aggressive pacing to be a strong predictor of subsequent slowing. That the increased risk of hitting the wall, in the years before and after a PB is greater among male runners is also consistent with the tendency of males to engage in more risky pacing as reported by Hubble et al. [53]. Of course pacing may also be impacted by the topology and conditions of a particular course and event. Recent work by Oficial-Casado et al. [51] shows that the pacing profiles associated with different marathons differ based on finish-time categories and it is plausible to conclude that some courses may be more susceptible to runners hitting the wall than others.

A second novel contribution of this work concerns the finish-time costs associated with hitting the wall. The existing literature remains largely silent on this feature of the phenomenon, perhaps because of the difficultly in determining what might have been a reasonable finish-time for a runner had they not hit the wall. Also, many past studies have focused on incidents of hitting the wall in isolated races or a small set of races [1, 11, 14, 16], rather than by tracking the performance of runners over an extended series of races. The scale of the dataset used in this study makes it feasible to consider a runner’s (partial) marathon history and, as such, provides an opportunity to use an estimate of runner’s recent PB as a benchmark against which to evaluate the cost of their hitting the wall. Finding that faster runners experience a greater *HTW Cost* is surprising at first, because it suggests faster runners slow more when they hit the wall. However, since *HTW Distance* and *HTW Slowdown* increase with PB time (Fig 5d and 5f), this means that the higher HTW costs for faster runners must be due to proportionally faster PB times rather than slower HTW times. This is consistent with research highlighting sub-optimal pacing by slower runners [42] in general, and may indicate that, all other things being equal, the PBs of slower runners are less optimal than the PBs of faster runners, even allowing for ability differences.

Although this paper highlights a well-known disparity between the proportion of male and female runners hitting the wall, the results also show that, when runners hit the wall, they do so in a broadly similar manner with similar consequences. This of course speaks to a common mechanism underpinning the phenomenon, while the different proportions of male and females hitting the wall emphasises critical differences in their risk-taking behaviours, when it comes to pacing. In this regard at least, runners and coaches have the potential impose some level of control on whether a runner will hit the wall, by focusing on making better pacing decisions and by being aware of the increased pacing risk that exists, for males in particular, and for all runners when they are pursing a PB.

## Limitations

As with any study of this nature, there are a number of assumptions and limitations worth discussing. First and foremost, this work relies on a particular definition of hitting the wall that is purely based on in-race pacing. In reality, hitting the wall is a multi-factorial phenomenon, which reflects a complex set of interactions between training, fitness, pacing, nutrition, and race-day conditions, and, as such, the model used here cannot capture the full complexity of the phenomenon. Nevertheless, we propose that it is reasonable and useful to consider significant late-race slowing as a proxy for hitting the wall, as others have done [15]. Although not every single slowdown can be explained by the runner hitting the wall (e.g. under-training, injury, or simply “giving up” can provide alternative explanations), runners who do hit the wall can be expected to slow significantly. Certainly, this model can be improved by incorporating additional sources of data, such as heart-rate data, for example, which may facilitate more accurate judgements about whether a runner has hit the wall. Although such data was not available in our dataset, the increasingly widespread adoption of mobile devices, smart-watches, and wearable sensors [55, 56] has the capacity to generate large volumes of additional data (heart-rate, cadence, and power), which may be useful in this regard in the future [57, 58]. Already, the availability of such diverse sources of data is enabling several new types of health and fitness applications [59–63] and the emergence of powerful new machine learning techniques has been used to support a variety of related prediction and planning tasks in several sporting domains [64–73]

It is also worth noting that the model of the wall analysed here is defined by a pair of parameters—degree of slowdown and length of slowdown—with specific values—0.25 and 5km, respectively—and it is reasonable to question whether the results would be different if different values had been chosen. We have considered several alternative sets of values and, within reasonable levels of tolerance, there is no material change to the nature of the results as presented. These additional results are available as S1–S14 Figs.

Another limitation of the approach is that, although we have collected a large corpus of race records, it does not provide a complete account of the marathon history for many, if not most, runners. This undermines our estimation of runner ability, because it relies on the fastest *available* finish-time for a runner during a *recent* race as their recent PB time estimate. Their true recent PB time may be associated with a race that is not in our dataset and thus we can expect our PB estimates to underestimate (be slower than) a runner’s true PB. Thus our estimates of the cost of hitting the wall may also underestimate the true cost of hitting the wall. However, because the dataset used in this study is based on many of the largest marathons in the world we propose that it is likely to provide a reasonably accurate estimate of the PB times of runners, because runners are more likely to train for, target, and achieve PBs at these landmark races. Even if the PBs used here are not always true PBs, it is likely that they will correlate closely with true PBs and, as such, the trends observed, and the relative differences found, can be expected to be reasonable.

The dataset is also limited in terms of the pacing precision that it provides. For instance, the availability of 5km segment times/pacing limits the granularity with which we can explore the nature of the wall. Using more fine-grained pacing data, such as that collected by smartwatches or GPS apps, it will be possible to provide much more fine-grained insights into what it means when runners hit the wall; see for example [15]. A similar lack of precision exists for much of the age data that is provided. Although some marathons provide access to precise age (or year of birth) data, most use age ranges. This limits the precision of our age-related analyses. Nevertheless, the results suggest that, when it comes to hitting the wall, age is less important than sex or ability and, as such, it is unlikely that more fine-grained age data would reveal results that are significantly different from those reported.

## Conclusions

We have described the results of a large-scale data analysis, focused on the marathon race records of recreational runners in big-city marathons, in order to better understand when and how runners hit the wall. The key findings include:

1. A greater proportion of male runners hit the wall, compared with female runners, and the likelihood of hitting the wall is strongly correlated with the PB times of runners in the 180–300 minute range.
2. The likelihood of hitting the wall increases in the years directly before and after a runner achieves a new personal-best time, regardless of age or ability.
3. When runners hit the wall they tend to do so in a broadly similar manner, although male runners slow for slightly longer, and by more, than female runners.
4. The finish-time cost of hitting the wall, relative to PB times, is greater for faster runners, primarily because they achieve *relatively* faster PB times, compared to slower runners.

Despite the limitations inherent in this work—a purely pacing-based definition of the wall with limited pacing precision (5km splits) and age precision (age ranges) and a finite and incomplete dataset of race records—the work is expected to be of interest to sport scientists, coaches, and runners alike, especially in the area of recreational marathon running.

## Supporting information

S1 Table List of marathon data sources. A table containing all of the URLs of the marathon web-sites used as a source of data for this study. Typically marathons maintain an archive of past race results either accessible directly via a web interface linked to from the main marathon website, or accessible via the websites of third-party timing services. A minority of marathons provide access to data which can be downloaded in bulk, while a majority provide access to their results via a search-based interface and in a page-based format. The data obtained used in this study were obtained directly from result archives between 2015 to 2019.
(DOCX)

S1 Datasets The raw datasets and statistics for each analysis result graph. Each individual result graph is associated with 4 different comma-separated files: (i) *Raw*—the (anonymised) *raw* data behind the means and standard deviations used for a particular result graph; (ii) *Paired*—the *paired* statistical significance results; (iii) *Successive Male*—the statistical significance results to compare *successive* groups (age and ability) for *male* runners; and (iv) *Successive Female*—the corresponding results for the statistical significance tests to compare *successive* groups (age and ability) of *female* runners.
(ZIP)

S1 Fig HTW proportions for male and female runners by age range. (TIF)

S2 Fig HTW proportions for male and female runners by ability level. (TIF)

S3 Fig HTW start (km) for male and female runners by age range. (TIF)

S4 Fig HTW start (km) for male and female runners by ability level. (TIF)

S5 Fig HTW distance (km) for male and female runners by age range. (TIF)

S6 Fig HTW distance (km) for male and female runners by ability level. (TIF)

S7 Fig HTW slowdown for male and female runners by age range. (TIF)

S8 Fig HTW slowdown for male and female runners by ability level. (TIF)

S9 Fig HTW time (mins) for male and female runners by age range. (TIF)

S10 Fig HTW time (mins) for male and female runners by ability level. (TIF)

S11 Fig HTW cost (mins) for male and female runners by age range. (TIF)

S12 Fig HTW cost (mins) for male and female runners by ability level. (TIF)

S13 Fig Relative HTW cost (mins) for male and female runners by age range. (TIF)

S14 Fig Relative HTW cost (mins) for male and female runners by ability level. (TIF)
