# Supplementary material for: How recreational marathon runners hit the wall: A large-scale data analysis of late-race pacing collapse in the marathon
Source: PLoS One. 2021 May 19;16(5):e0251513. doi: 10.1371/journal.pone.0251513 (PMC8133477; doi:10.1371/journal.pone.0251513)
Supplement: S1 Table — A table containing all of the URLs of the marathon web-sites used as a source of data for this study. Typically marathons maintain an archive of past race results either accessible directly via a web interface linked to from the main marathon website, or accessible via the websites of third-party timing services. A minority of marathons provide access to data which can be downloaded in bulk, while a majority provide access to their results via a search-based interface and in a page-based format. The data obtained used in this study were obtained directly from result archives between 2015 to 2019. (DOCX) [file pone.0251513.s001.docx]

S1 Table. The marathon result archives used to provide the race records for this study.

| **City** | **URL** |
| --- | --- |
| Amsterdam | https://www.tcsamsterdammarathon.eu/results |
| Athens | https://www.athensauthenticmarathon.gr/site/index.php/en/results-en |
| Aukland | https://results.timingsports.com/aucklandmarathon/2019 |
| Austin | https://youraustinmarathon.com/results/ |
| Barcelona | https://www.zurichmaratobarcelona.es/eng/resultados_e/2019 |
| Berlin | https://www.bmw-berlin-marathon.com/en/impressions/statistics-and-history/results-archive/ |
| Boston | https://www.baa.org/races/boston-marathon/results/search-results |
| Chicago | https://www.chicagomarathon.com/runners/race-results/ |
| Cologne | https://koeln-marathon.de/en/results/ |
| Copenhagen | https://copenhagenmarathon.dk/en/resultatarkiv/ |
| Dubai | https://dubai.mikatiming.de/2020/?pid=search&pidp=start |
| Eindhoven | https://www.marathoneindhoven.org/info/previous-editions/results-2017/ |
| Frankfurt | https://live.frankfurt-marathon.com/2019 |
| Gold Coast | https://goldcoastmarathon.com.au/past-results/ |
| Hamburg | https://www.haspa-marathon-hamburg.de/en/results/ |
| Helsinki | https://www.helsinkimarathon.fi/en/results/ |
| Honolulu | https://www.honolulumarathon.org/results |
| Houston | https://www.chevronhoustonmarathon.com/participants/results/ |
| Los Angeles | https://live.xacte.com/lamarathon/ |
| London | https://www.virginmoneylondonmarathon.com/en/results/race-results |
| Madrid | https://edprocknrollmadrid.com/results/?lang=en |
| Melbourne | https://melbournemarathon.com.au/results/ |
| Mexico | https://www.maratoncdmx.com |
| Moscow | https://results.chronotrack.com/event/results/event/event-32804 |
| New York | https://www.nyrr.org/tcsnycmarathon/Results/Race-Results |
| Oslo | https://oslomaraton.no/en/resultater/ |
| Paris | https://resultscui.active.com/events/schneiderelectricmarathondeparis2019 |
| Prague | https://www.runczech.com/srv/www/qf/en/ramjet/results/list?&page=1&per_page=15&yearFilter= |
| Rome | https://www.endu.net/en/events/maratona-internazionale-roma/results |
| Rotterdam | https://www.nnmarathonrotterdam.org/info/previous-editions/edition-2019/ |
| Singapore | https://singaporemarathon.com/race/results/ |
| Stockholm | https://registration.marathongruppen.se/ResultList.aspx?LanguageCode=en&RaceId=51 |
| Sydney | https://sydneyrunningfestival.com.au/pastresults/ |
| Tokyo | https://www.marathon.tokyo/en/about/past/ |
| Valencia | https://www.valenciaciudaddelrunning.com/en/marathon/previous-editions-marathon/ |
| Vienna | https://www.vienna-marathon.com/?lang=en&surl=cd162e16e318d263fd56d6261673fe72#goto-result |
| Warsaw | https://maratonwarszawski.com/en/aktualnosci/official-results-of-41st-pzu-warsaw-marathon/ |
| Washington | https://www.marinemarathon.com/results/marathon |

The raw data comes in a variety of formats. Typically, marathons maintain an archive of past race results accessible directly via a web interface linked to from the main marathon website or accessible via the websites of third-party timing services. A minority of marathons provide access to data which can be downloaded in bulk.
