# Supplementary figures and images for: How recreational marathon runners hit the wall: A large-scale data analysis of late-race pacing collapse in the marathon
Source: PLoS One. 2021 May 19;16(5):e0251513. doi: 10.1371/journal.pone.0251513 (PMC8133477; doi:10.1371/journal.pone.0251513)

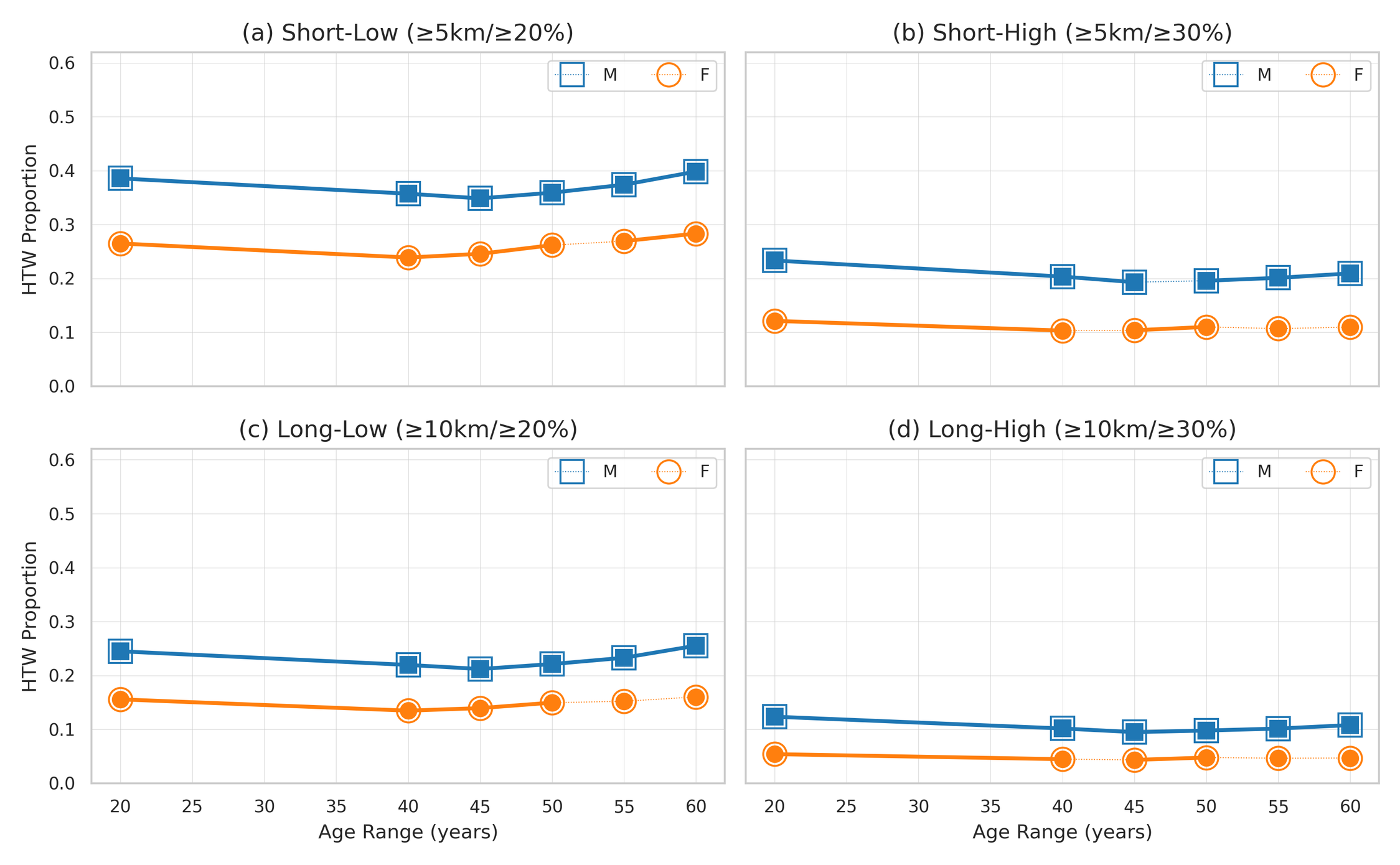

Supplement: S1 Fig — (TIF) [file pone.0251513.s003.tif]

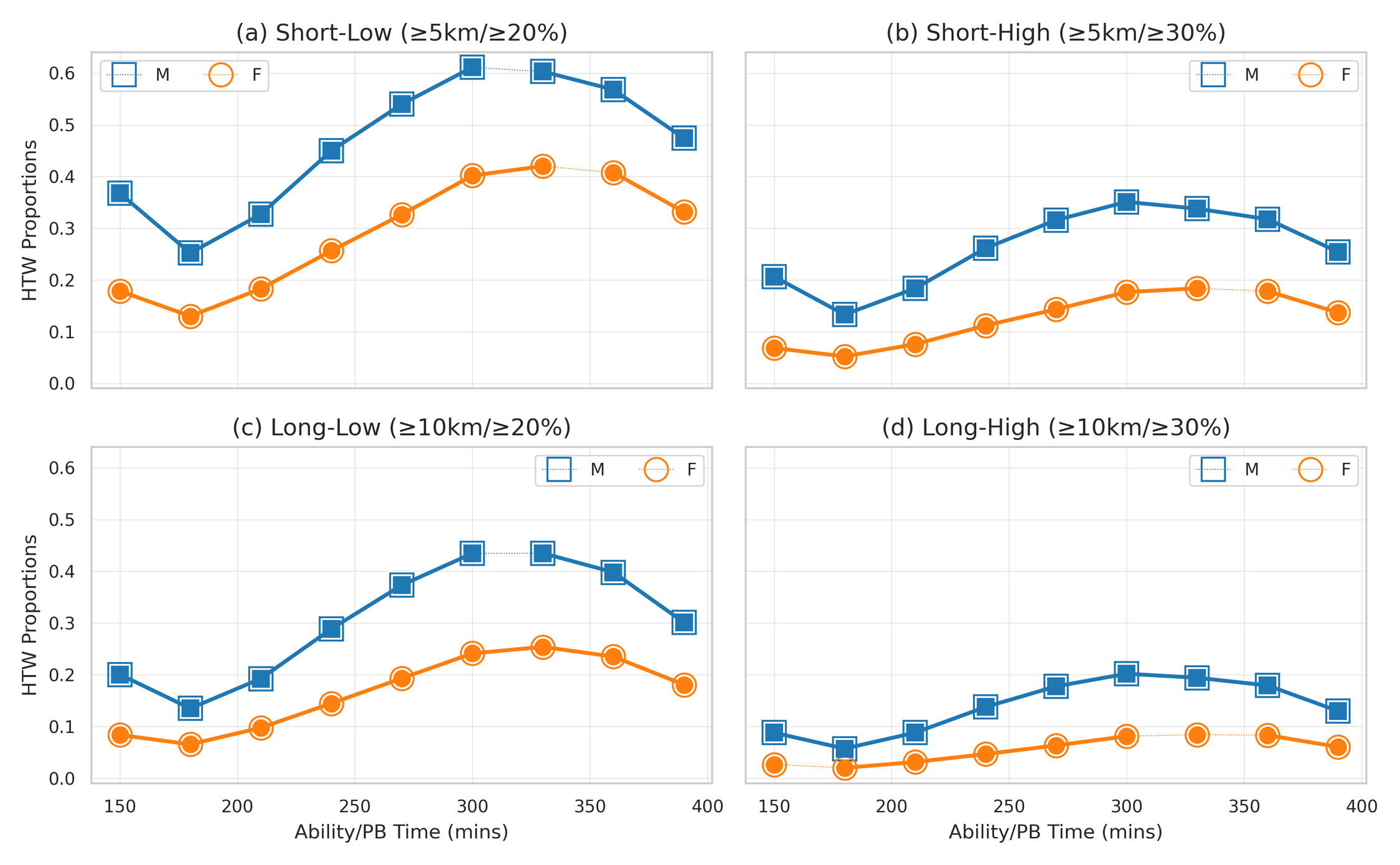

Supplement: S2 Fig — (TIF) [file pone.0251513.s004.tif]

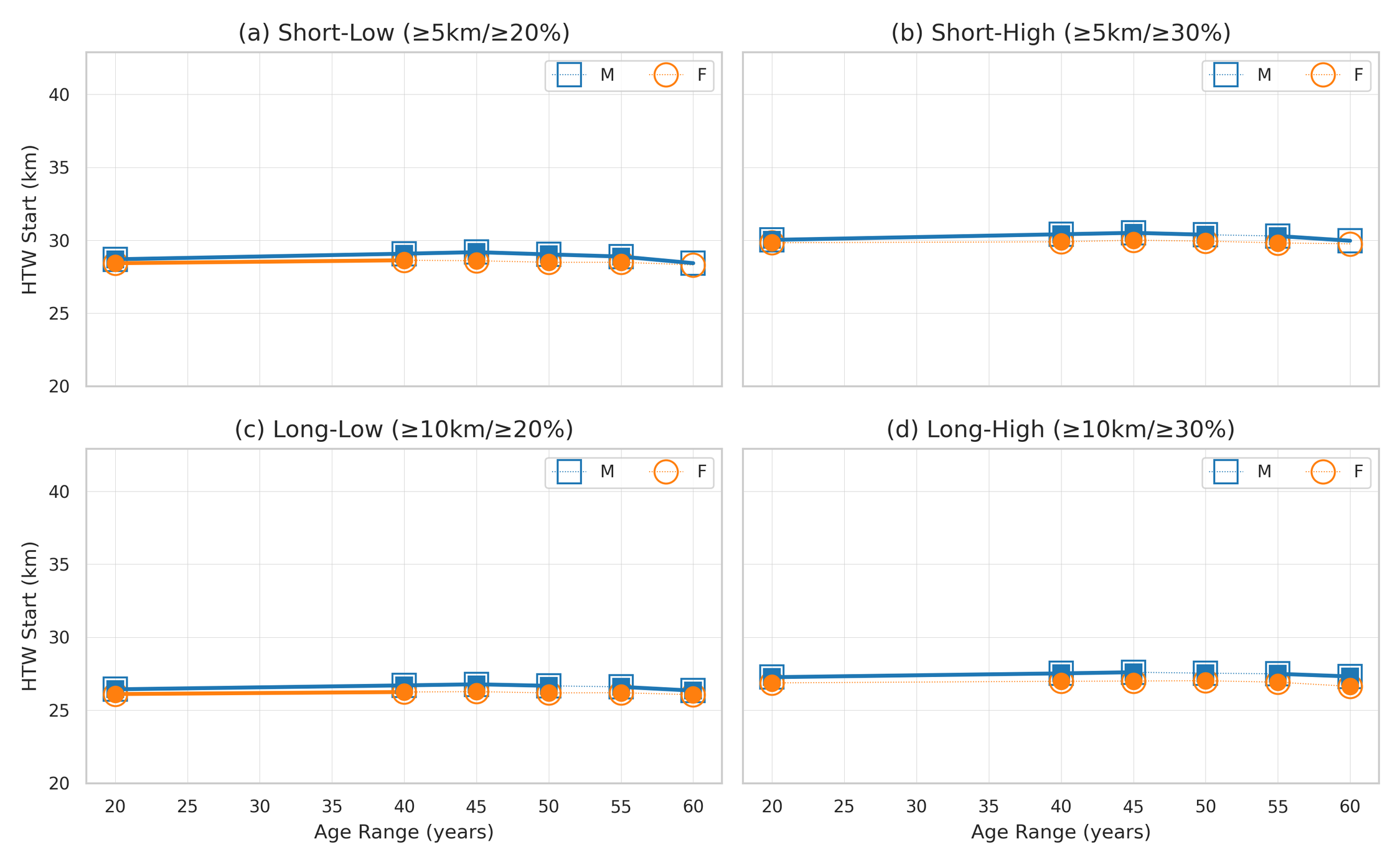

Supplement: S3 Fig — (TIF) [file pone.0251513.s005.tif]

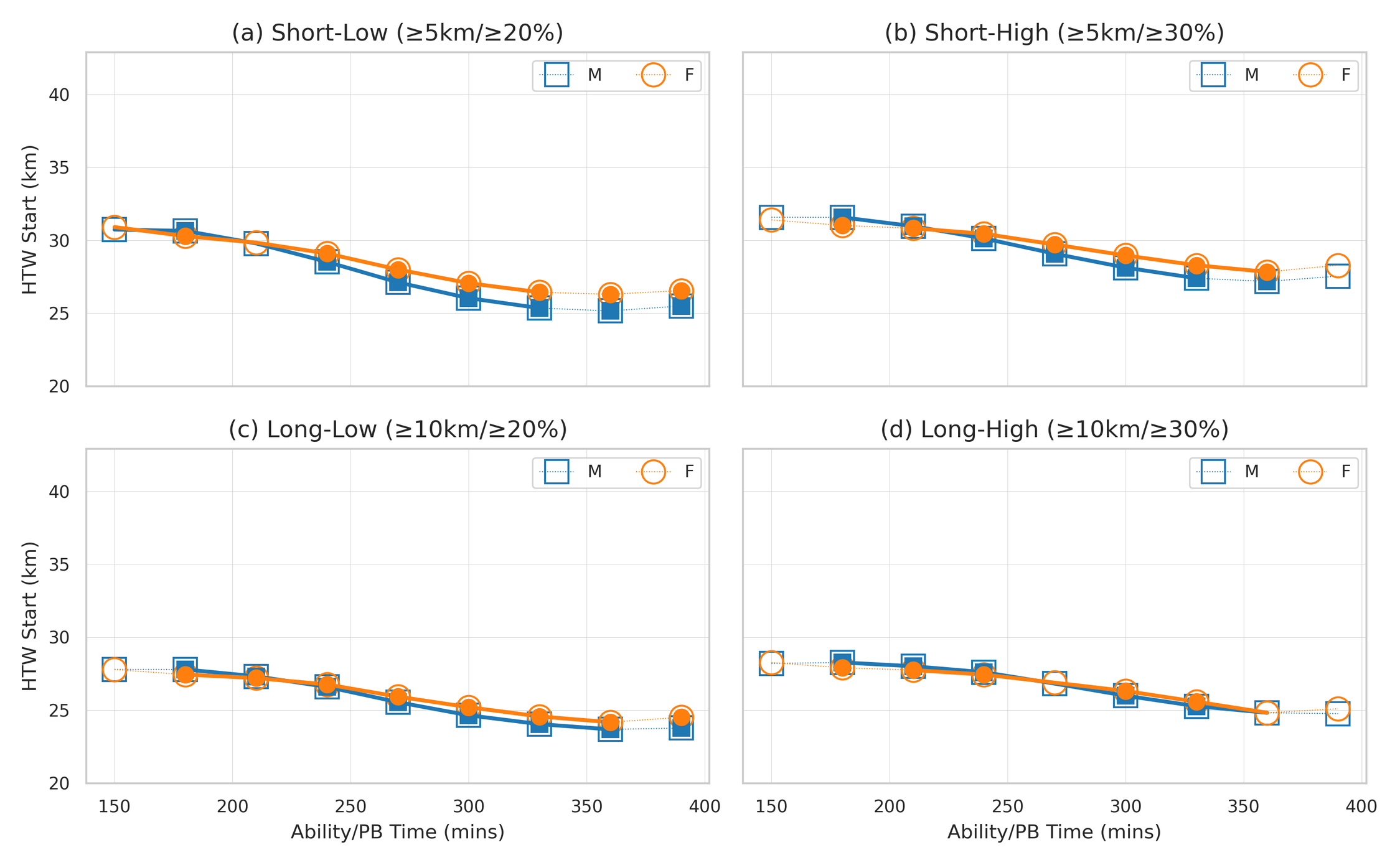

Supplement: S4 Fig — (TIF) [file pone.0251513.s006.tif]

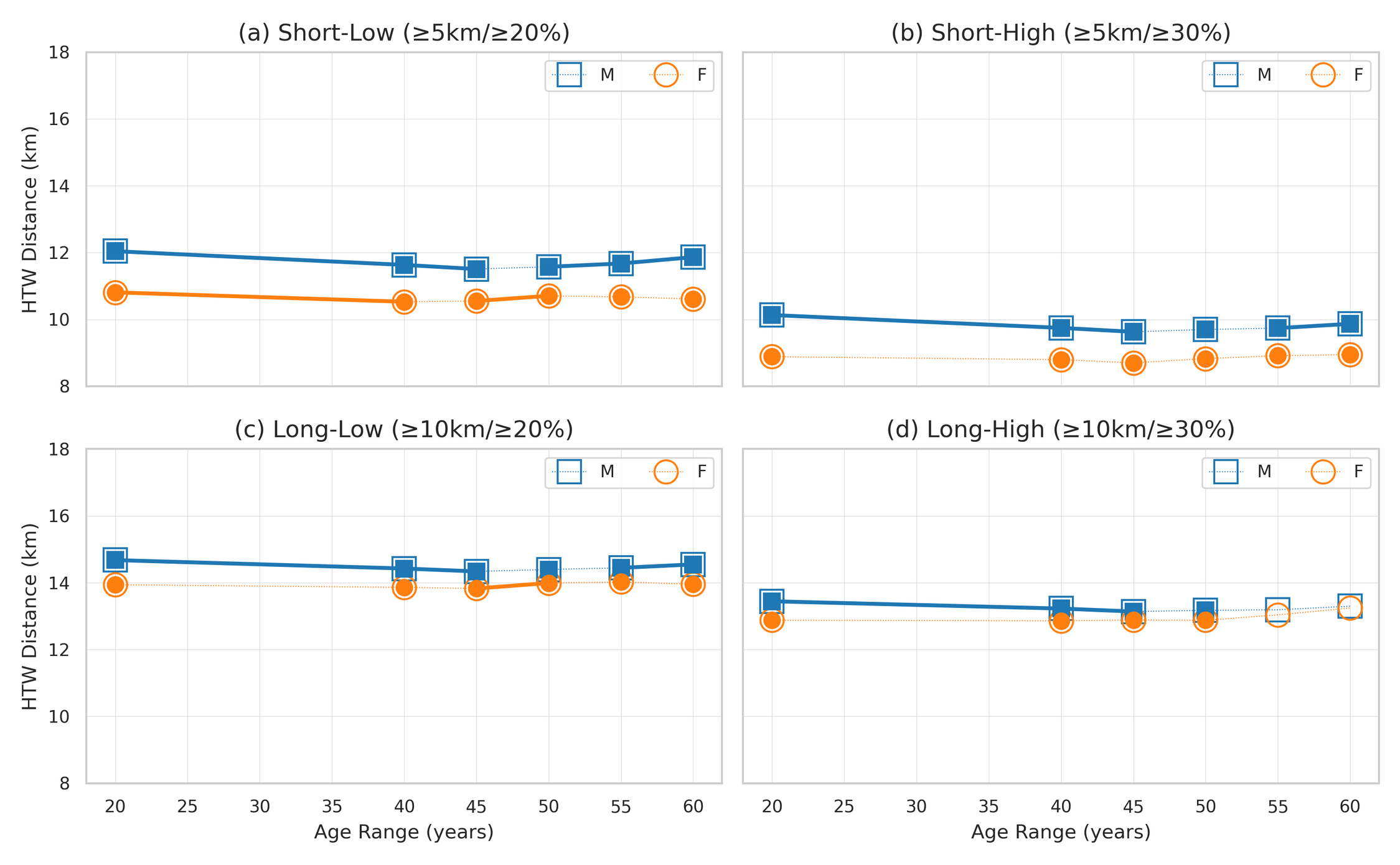

Supplement: S5 Fig — (TIF) [file pone.0251513.s007.tif]

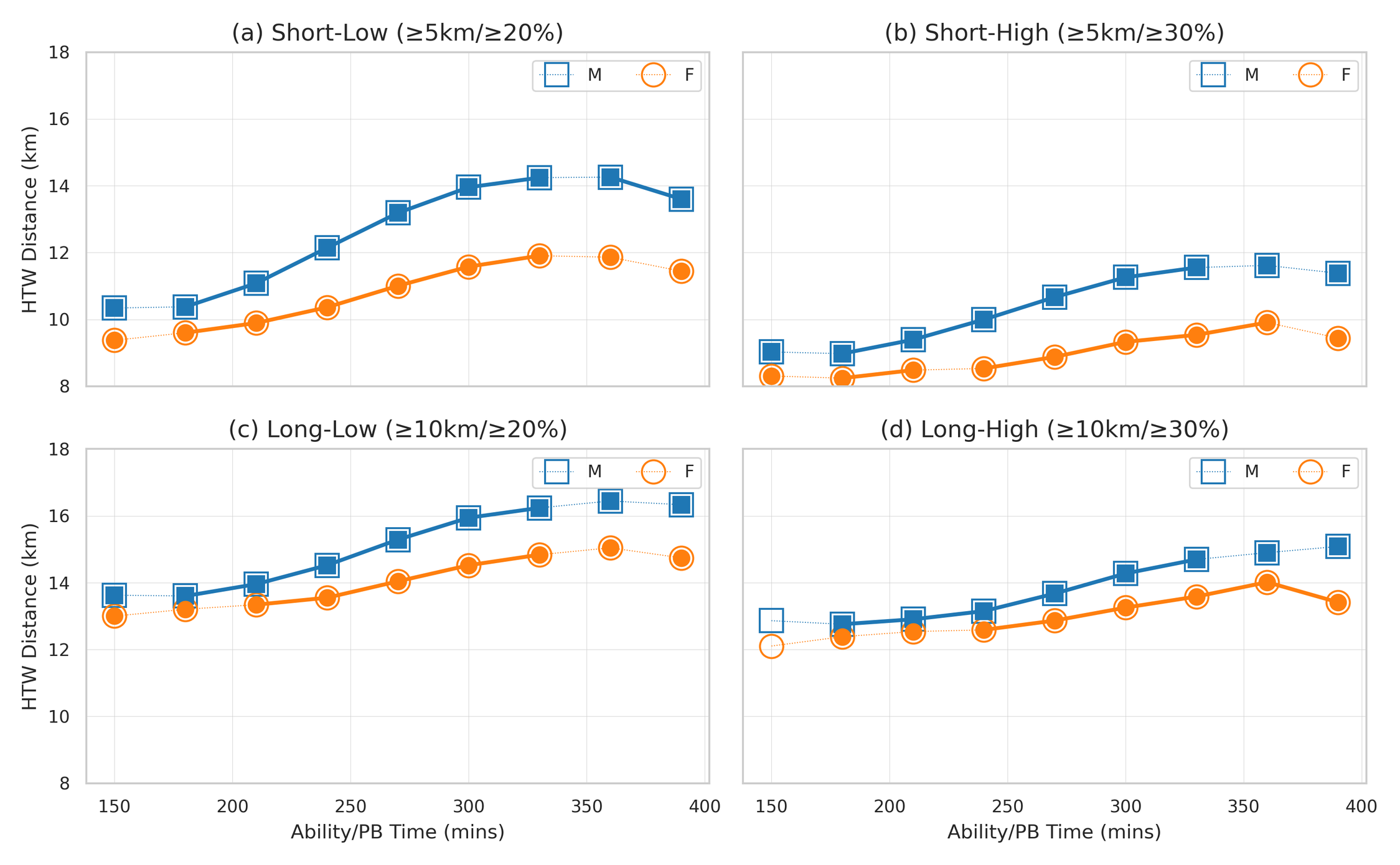

Supplement: S6 Fig — (TIF) [file pone.0251513.s008.tif]

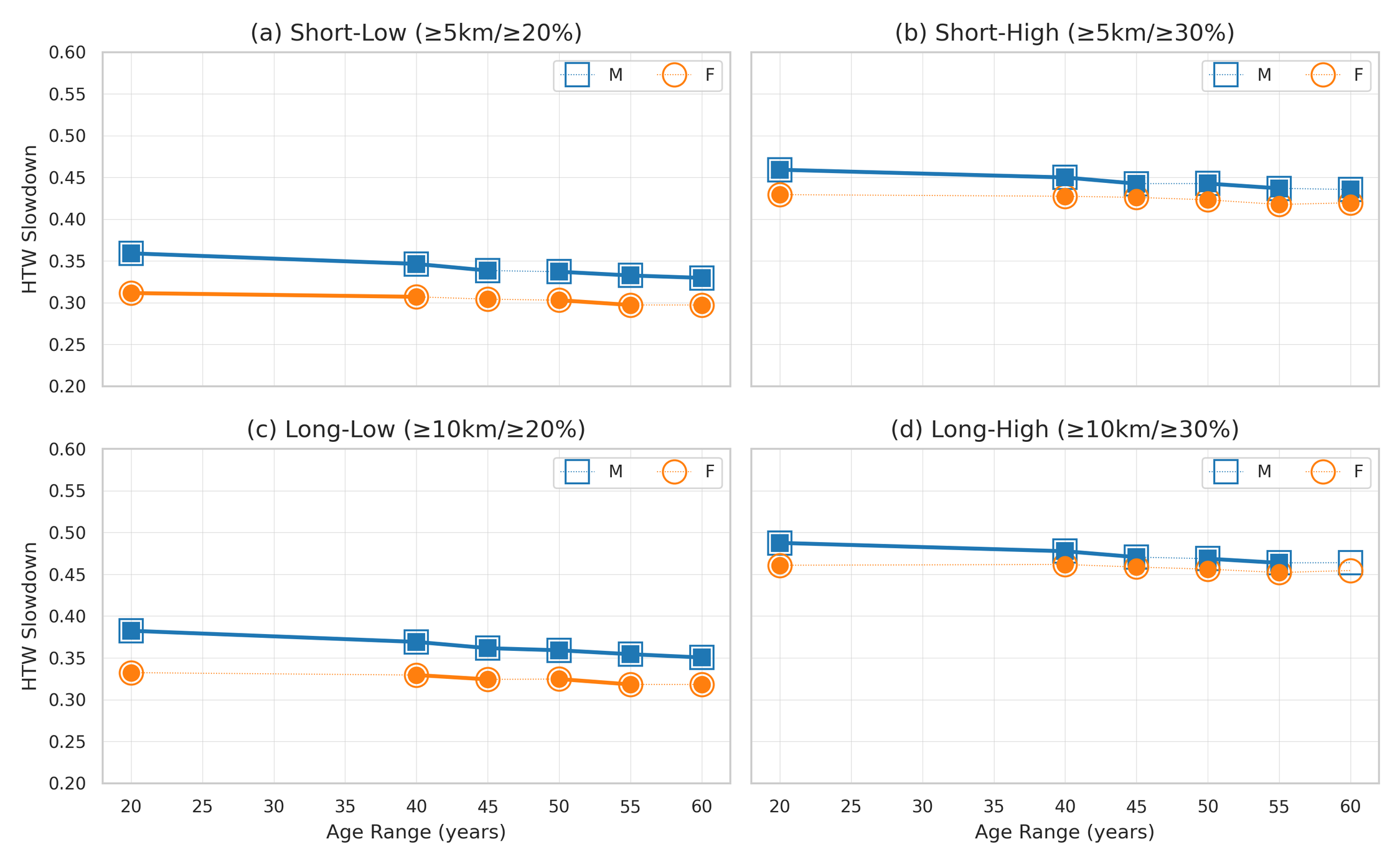

Supplement: S7 Fig — (TIF) [file pone.0251513.s009.tif]

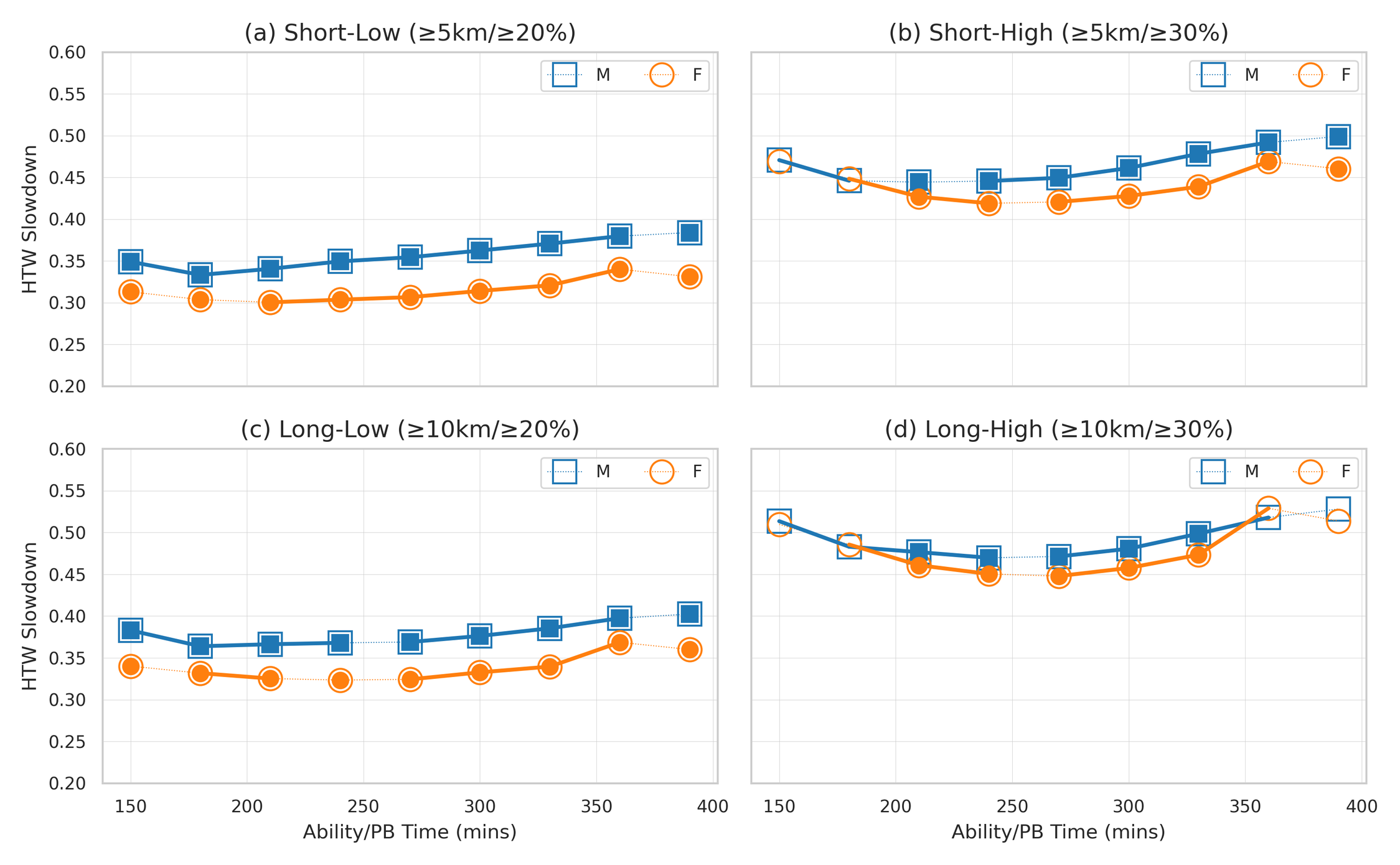

Supplement: S8 Fig — (TIF) [file pone.0251513.s010.tif]

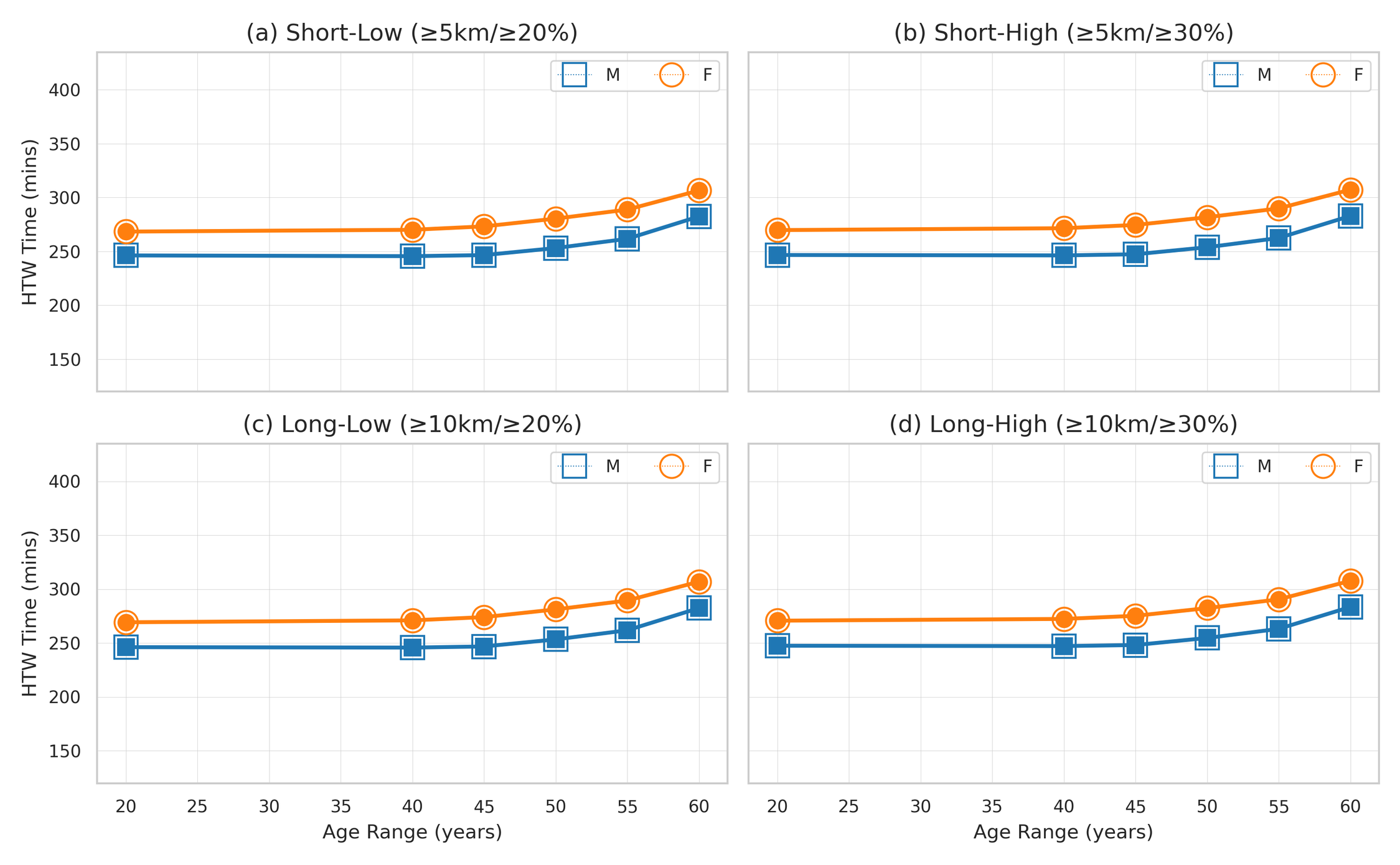

Supplement: S9 Fig — (TIF) [file pone.0251513.s011.tif]

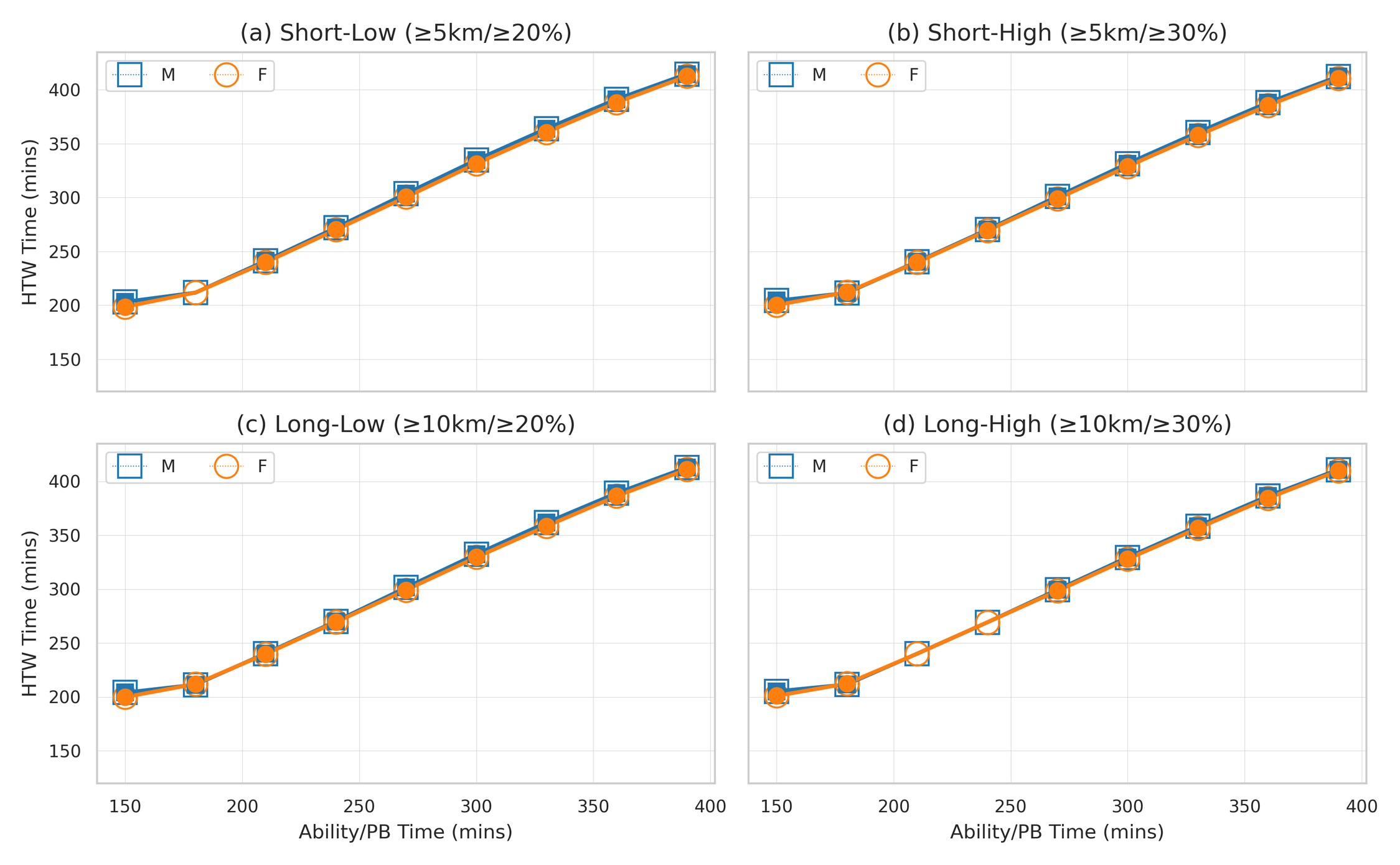

Supplement: S10 Fig — (TIF) [file pone.0251513.s012.tif]

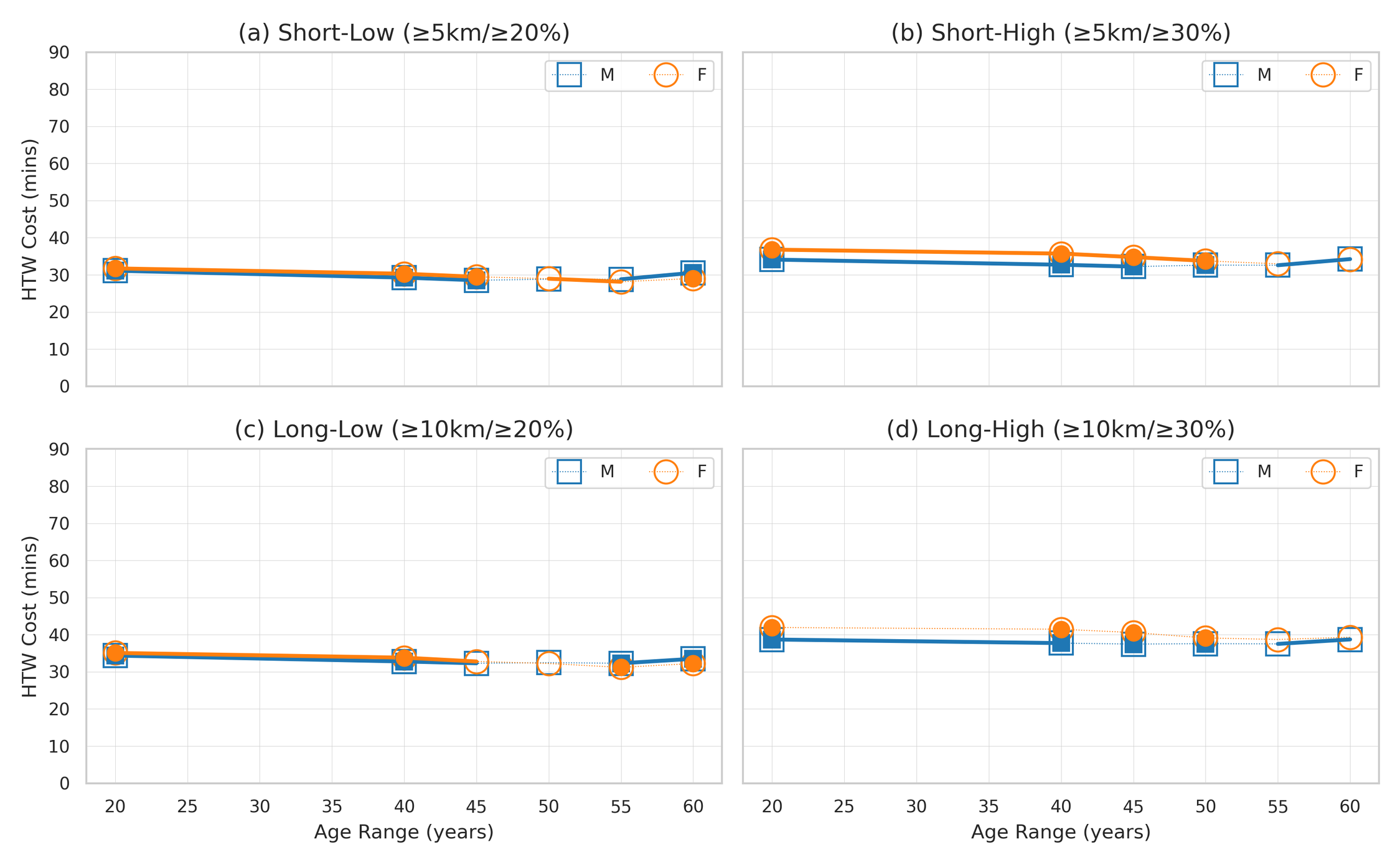

Supplement: S11 Fig — (TIF) [file pone.0251513.s013.tif]

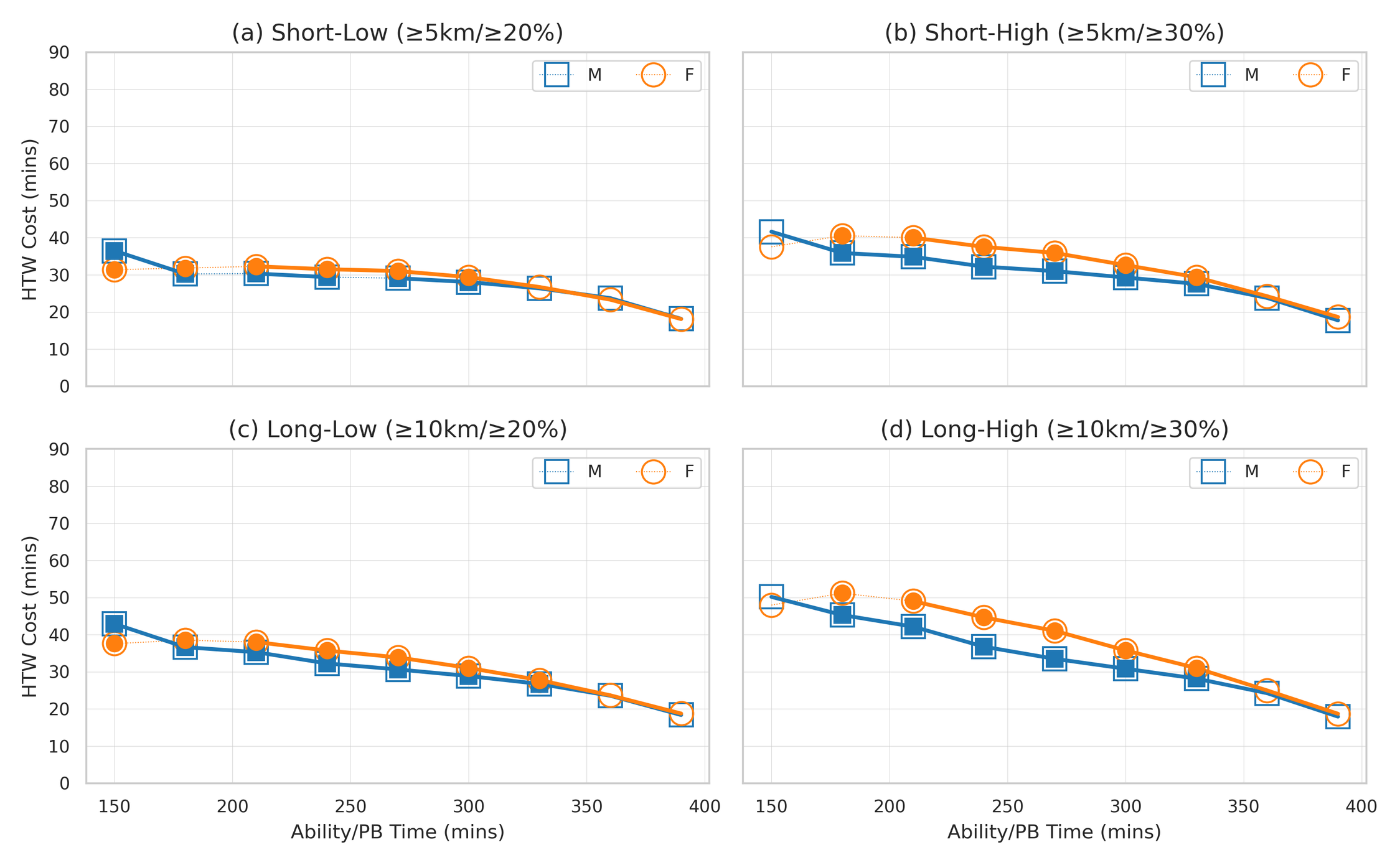

Supplement: S12 Fig — (TIF) [file pone.0251513.s014.tif]

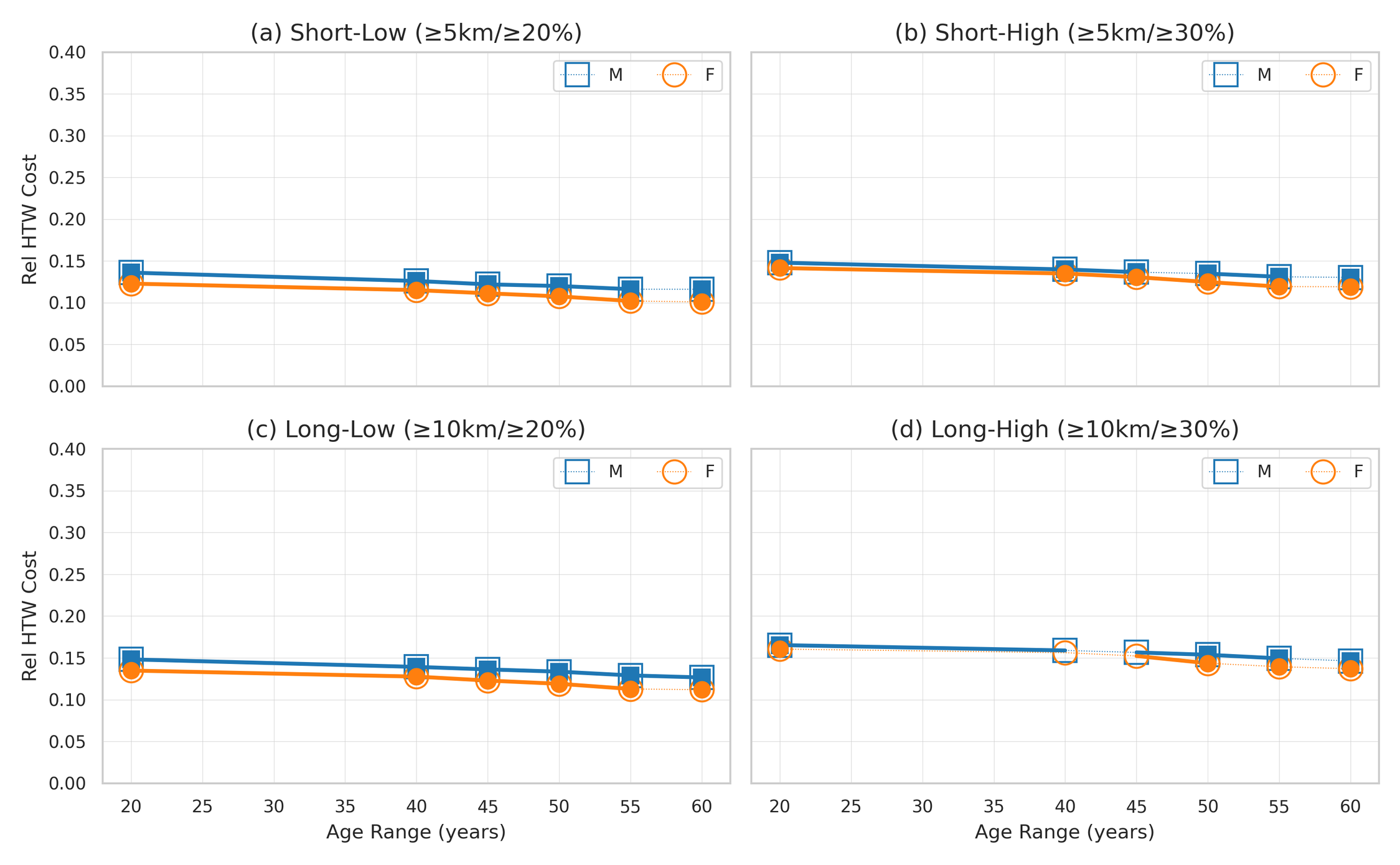

Supplement: S13 Fig — (TIF) [file pone.0251513.s015.tif]

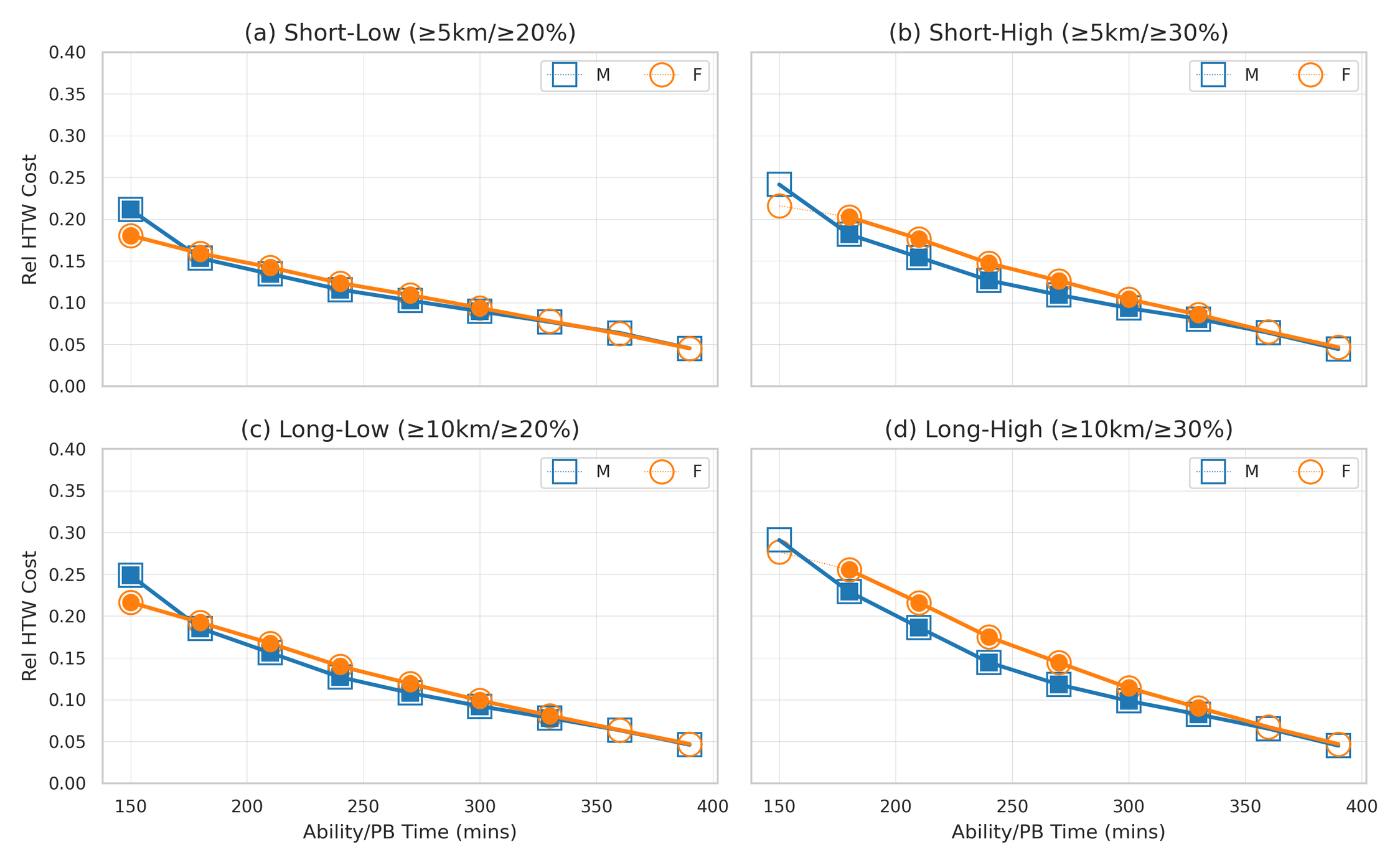

Supplement: S14 Fig — (TIF) [file pone.0251513.s016.tif]
